# Manipulating Google’s Knowledge Graph Box to Counter Biased Information Processing During an Online Search on Vaccination: Application of a Technological Debiasing Strategy

**DOI:** 10.2196/jmir.5430

**Published:** 2016-06-02

**Authors:** Ramona Ludolph, Ahmed Allam, Peter J Schulz

**Affiliations:** ^1^ Institute of Communication and Health Faculty of Communication Sciences University of Lugano (Università della Svizzera italiana) Lugano Switzerland; ^2^ Department of Pathology Yale University School of Medicine New Haven, CT United States

**Keywords:** search engine, online health information search, vaccination, debiasing, search behavior, health communication, information processing, information seeking

## Abstract

**Background:**

One of people’s major motives for going online is the search for health-related information. Most consumers start their search with a general search engine but are unaware of the fact that its sorting and ranking criteria do not mirror information quality. This misconception can lead to distorted search outcomes, especially when the information processing is characterized by heuristic principles and resulting cognitive biases instead of a systematic elaboration. As vaccination opponents are vocal on the Web, the chance of encountering their non‒evidence-based views on immunization is high. Therefore, biased information processing in this context can cause subsequent impaired judgment and decision making. A technological debiasing strategy could counter this by changing people’s search environment.

**Objective:**

This study aims at testing a technological debiasing strategy to reduce the negative effects of biased information processing when using a general search engine on people’s vaccination-related knowledge and attitudes. This strategy is to manipulate the content of Google’s knowledge graph box, which is integrated in the search interface and provides basic information about the search topic.

**Methods:**

A full 3x2 factorial, posttest-only design was employed with availability of basic factual information (comprehensible vs hardly comprehensible vs not present) as the first factor and a warning message as the second factor of experimental manipulation. Outcome variables were the evaluation of the knowledge graph box, vaccination-related knowledge, as well as beliefs and attitudes toward vaccination, as represented by three latent variables emerged from an exploratory factor analysis.

**Results:**

Two-way analysis of variance revealed a significant main effect of availability of basic information in the knowledge graph box on participants’ vaccination knowledge scores (*F*_2,273_=4.86, *P*=.01), skepticism/fear of vaccination side effects (*F*_2,273_=3.5, *P*=.03), and perceived information quality (*F*_2,273_=3.73, *P*=.02). More specifically, respondents receiving comprehensible information appeared to be more knowledgeable, less skeptical of vaccination, and more critical of information quality compared to participants exposed to hardly comprehensible information. Although, there was no significant interaction effect between the availability of information and the presence of the warning, there was a dominant pattern in which the presence of the warning appeared to have a positive influence on the group receiving comprehensible information while the opposite was true for the groups exposed to hardly comprehensible information and no information at all. Participants evaluated the knowledge graph box as moderately to highly useful, with no significant differences among the experimental groups.

**Conclusion:**

Overall, the results suggest that comprehensible information in the knowledge graph box positively affects participants’ vaccination-related knowledge and attitudes. A small change in the content retrieval procedure currently used by Google could already make a valuable difference in the pursuit of an unbiased online information search. Further research is needed to gain insights into the knowledge graph box’s entire potential.

## Introduction

### Background

There is little doubt today that the Internet has revolutionized consumers’ information search [1]. One of people’s major motives for going online is the search for health-related information [2,3]. However, the Web does not only offer the opportunity to access an abundance of information from a broad variety of sources but also bears the peril of “getting lost in information” [4] and an increased risk of encountering misinformation [1]. This holds especially true for the topic of vaccination. Despite the large evidence base that proves vaccines’ safety and effectiveness [5-8], vaccination opponents continue spreading numerous myths online about immunization [9-11]. Their high visibility on the Web [10,12,13] might have contributed to vaccination being a controversially discussed topic with many people being concerned about potential side effects and hesitant to adhere to official vaccination recommendations [14-19].

General search engines play a major role when it comes to the visibility of a topic. Given that most people start their search with a general search engine like Google, Bing, or Yahoo [2,20,21], a search engine’s sorting and ranking criteria can directly influence the search outcomes [22-24]. In turn, websites can be designed to better meet those criteria and achieve higher visibility [25]. Yet, most consumers do not seem to be aware of the logic behind search engines’ retrieval algorithms [23,24]. An eye tracking experiment revealed, for instance, that participants trusted Google to rank search results according to their pertinence to the search query [24]. This led to a closer scrutiny of highly positioned search results, regardless of their actual relevance [24]. Another study found the sorting and ranking criteria to impact information seekers’ attitudes and knowledge [23]. More specifically, when the authors manipulated the ratio of pro- and antivaccination websites displayed by Google, a detrimental effect of a high share of antivaccination websites was detected [23]. In contrast, the complete absence of negative information resulted in higher knowledge and more favorable attitudes toward vaccination [23]. The high share of misleading or false information concerning vaccination on the Web thus bears a peril for health information seekers [11], especially if the websites fulfill a search engine’s conditions to get a high rank on the results pages.

One explanation for consumers’ neglect of the logic behind the results displayed by a search engine and the associated suboptimal search outcomes could be a lack of systematic information processing. Instead of systematically elaborating on the search and its results, online information seekers seem to apply heuristic principles [24,26-28]. Whereas systematic information processing is characterized by a “thorough, in-depth, complete, and well-advised processing of all given information,” heuristic processing can be described as “relying on cues that signal truth, quality, or validity” [28]. Especially experienced Web users, as compared to inexperienced ones, were found to process information rather heuristically when using a search engine [28]. A heuristic can be defined as a “cognitive shortcut that relies on little information and modest cognitive resources” and often results in satisfying outcomes [29]. So-called fast and frugal heuristics exploit the composition of a certain environment in a prompt and relatively effortless manner [30] and might thus help experienced information seekers to quickly scan a search engine’s results without investing too much cognitive effort [24].

However, the ignorance of crucial parts of information can also result in cognitive biases [29,31,32], a phenomenon that was primarily demonstrated in the classic experiments by Tversky and Kahneman [31]. Cognitive biases are understood as “systematic error[s] in judgment and decision-making…which can be due to cognitive limitations, motivational factors, and/or adaptations to natural environments” [29]. In the context of online information seeking via a general search engine, the use of heuristic principles and accompanied reduction of complexity might lead to derogated search outcomes such as a biased processing or interpretation of the results [23,24]. Eventually, this can have negative effects on people’s subsequent judgment and decision making if it is based on the search results [23].

### Previous Studies

Indeed, several studies report the occurrence and detrimental effects of search-related cognitive biases [23,33-35]. Findings of one study demonstrated that clinicians experience cognitive biases such as anchoring, exposure, or order bias during the online information search [33]. More specifically, participants’ prior beliefs about the search topic, the mere time they spent on processing the information, and the position where the information was placed in the retrieval system influenced people’s postsearch decisions [33]. Moreover, it was found that health information seekers who start their search with a “strong specific hypothesis” about the search topic tend to focus their search on the verification of this belief and are inclined to interpret the search results as supporting their initial idea [34]. These tendencies are also known as positive hypothesis testing and confirmation bias and were supported by another study. There, the author demonstrated that participants “were very prone to positive hypothesis testing when they searched for health information using a popular search engine” [35].

To overcome these unwanted effects of cognitive biases, debiasing interventions were designed and tested. Debiasing techniques aim at eliminating, reducing, or reversing detrimental effects on judgment and decision making caused by cognitive biases [36-40]. Taking into account the underlying causes for the occurrence of those biases, one can differentiate between motivational, cognitive, and technological debiasing techniques [39]. Motivational strategies involve the promise of incentives or holding people accountable for their judgment in order to increase their motivation [39]. Cognitive and technological strategies are based on the assumption that intuitively applied decision strategies are imperfect, “but that they can be replaced by strategies that approach normative standards” [39]. For instance, “consider the opposite” could be regarded as a cognitive strategy [39]. Technological strategies, in turn, comprise the provision of external tools and techniques to improve the decision environment [39].

The application of technological debiasing strategies seems to be especially promising in the context of online health information seeking since the Web setting offers new opportunities to integrate those external tools [37]. This renders a long and resource-intense debiasing of the individual unnecessary but allows for a central change of the information seekers’ environment. Although the emphasis on the importance of an individual’s environment was mainly stimulated by the fast and frugal heuristics program [29], it also initiated the development of new technological debiasing approaches. One example is the implementation of a search interface that urges the user to order the search results in a particular manner [41]. This change of the search environment was shown to have a debiasing effect [41]. Another author tested two further technological debiasing techniques in the context of online health information seeking, namely recommendation and incorporation [35]. The recommendation strategy implied the automated suggestion of additional information by the search engine based on the entered keywords [35]. However, this strategy did not lead to a significant reduction of participants’ positive hypothesis testing [35]. In contrast, the incorporation strategy turned out to be an effective debiasing technique. It involved the configuration of a search engine that automatically built in search results with an opposite meaning than the ones entered by the participants [35]. For instance, when consumers entered “hypertension,” search results referring to “low blood pressure” were also displayed [35].

In sum, implemented technological debiasing interventions either involved the design of a specific search engine, which is complex and time consuming [41], or were not successful in reducing the undesired effects [35], or rather “outwitted” the information seekers instead of transparently countering their biased information processing [35]. Hence, the existing debiasing attempts call for new ideas and further research in implementing technological debiasing strategies. Drawing on the assumed potential of changing environmental structures, this study seeks to test the debiasing effects of a knowledge graph box. A knowledge graph box is a little box containing a short summary related to the search topic, displayed on the right upper side of the screen after the keywords of the search are entered. It was introduced by Google in mid-2012 [42] with the goal of providing semantic information related to the search topic gathered from various knowledge bases (eg, Freebase/Wikidata). In our study, the knowledge graph box was visible to participants occupied with an information search task as part of an online experiment intended to mitigate people’s deficient processing of health information found via Google.

### Objective and Hypotheses

Our aim was to design a debiasing intervention that would interrupt information seekers’ biased information processing and lead to a more systematic scrutinizing of search results, finally resulting in more knowledge and better attitudes toward vaccination. The design of the intervention was based on two lines of research. First, we followed the assumptions of technological debiasing by changing the search environment. More specifically, we added a manipulated version of Google’s knowledge graph box to the search interface. Second, the knowledge graph box’s content was designed in accordance with the assumption that a message’s effectiveness should mainly depend on its content, as opposed to receiver or source characteristics [43].

The content was divided into two parts that could possibly interfere with the biased information search, namely making (hardly) comprehensible basic factual information about vaccination available and a warning message. In general, we assumed the content of the knowledge graph box would serve as an implicit reference for the subsequently explored search results.

Concerning the basic factual information, two definitions of vaccination were provided. These were neutral in tone to avoid an unintended biasing of participants or reactance. However, they represented a different level of comprehensibility. Several experiments in the context of medical information revealed that comprehensible scientific content is more persuasive and receives stronger agreement from laypeople than does incomprehensible content [44,45]. Comprehensibility refers to the extent to which a health information seeker would quickly and easily understand what they read (see also [44]) and was operationalized in two ways. The first indicator for comprehensibility was the readability of the text as assessed by a readability formula such as the Flesch-Kincaid Reading Ease, where higher scores point to easier readability [46,47]. For the comprehensible definition, the formula provided a score of 23.7, whereas the hardly comprehensible one received a score of only -12.1 [47]. The second indicator for comprehensibility was the number of unexplained technical terms [44]. The hardly comprehensible definition used more unexplained medical terms such as “antigenic material,” “adaptive immunity,” or “pathogen,” whereas the comprehensible definition linked common language explanations to the medical expressions. Extending the results of previous research on the persuasive effects of comprehensibility to our context, we expected participants exposed to the comprehensible version of the basic information to show a higher postsearch vaccination knowledge level (Hypothesis 1) and more favorable attitudes toward vaccination (Hypothesis 2) as compared to the groups receiving hardly comprehensible information or no basic information at all.

As a second feature of the knowledge graph box, a warning message was intended to serve as a quality alert to the health information seekers. This is based on research from the area of consumer psychology where participants were found to detect manipulative practices more likely after they had been explicitly warned about their occurrence [48]. To avoid unintended reactions on the participants’ side, we refrained from an explicit warning about vaccination opponents’ views. Instead, a neutral warning about the existence of false or misleading information about vaccination on the Web was used. This quality alert was predicted to interrupt people’s heuristic use of the search engine since it is a new and unexpected search element [28,49] pointing to the need of a thorough inquiry of the search results [48]. The subsequent shift to a more systematic search should then lead to an elaborate information processing characterized by a closer scrutinizing of the search results’ quality. Accordingly, we hypothesize that the warning in the knowledge graph box will eventually be associated with higher postsearch knowledge levels (Hypothesis 3) and a more positive attitude toward vaccination (Hypothesis 4).

To achieve a comprehensive testing of the debiasing intervention, the two content-related factors were also combined. Here, we assumed that there could be an interaction effect of the availability of basic information (comprehensible vs incomprehensible) with the warning. However, as we could only speculate about its direction, we formulate Research Question 1: How do basic information and warning message interact to affect consumers’ vaccination-related knowledge and attitudes?

As this is, to our knowledge, the first study that investigates the impact of an experimentally manipulated knowledge graph box integrated into the search process, we are also interested in consumers’ perception and evaluation of it. Therefore, Research Question 2 asks: How is the knowledge graph box perceived and evaluated by the participants?

## Methods

### Design

In order to answer the research questions and test the hypotheses, a posttest-only online experiment was conducted. A full 3x2 factorial design was employed with the content of the knowledge graph box being varied. The first factor was availability of basic factual information on vaccination in the form of an evidence-based, medical definition (comprehensible vs hardly comprehensible vs not present) and the second factor was the presence or absence of a warning message about the occurrence of false information.

There were two versions of basic information that were extracted from two different sources, namely the World Health Organization (WHO) for the comprehensible version and Wikipedia for the less comprehensible version.

The warning stated that vaccination was a controversially discussed topic and that one would encounter false or misleading information about it on the Web. The warning did not support or elicit a standpoint toward the searched health topic.

In sum, the manipulations of the knowledge graph box resulted in the comparison of six different groups (see Table 1). The study was approved by the Ethical Committee of the University of Lugano, Switzerland. Further, all participants were asked for informed consent before taking part in the experiment.

**Table 1:** Experimental design and group allocation.

Knowledge graph box manipulation		Availability of basic information		
		Comprehensible basic information (WHO)	Hardly comprehensible basic information (Wikipedia)	No basic information
Warning of the presence of false information				
	No warning	Group 1	Group 2	Group 6 (Control group)
	Warning present	Group 4	Group 5	Group 3

### Manipulation of the Knowledge Graph Box

As we aimed for realistic results, the manipulation of the knowledge graph box mirrored the current practice of Google. It retrieves the semantic information from knowledge bases such as Wikidata [42], which acts as “central storage of structured data for Wikipedia” [50]. Hence, when searching for “vaccination” (as of January 2016), the knowledge graph box would display the same semantic information in a box right next to the search results that is also found in the corresponding Wikipedia article (see Multimedia Appendix 1 for a demonstration). Moreover, the knowledge graph box included a link to the original articles where the extracted information can be found.

**Figure 1:**
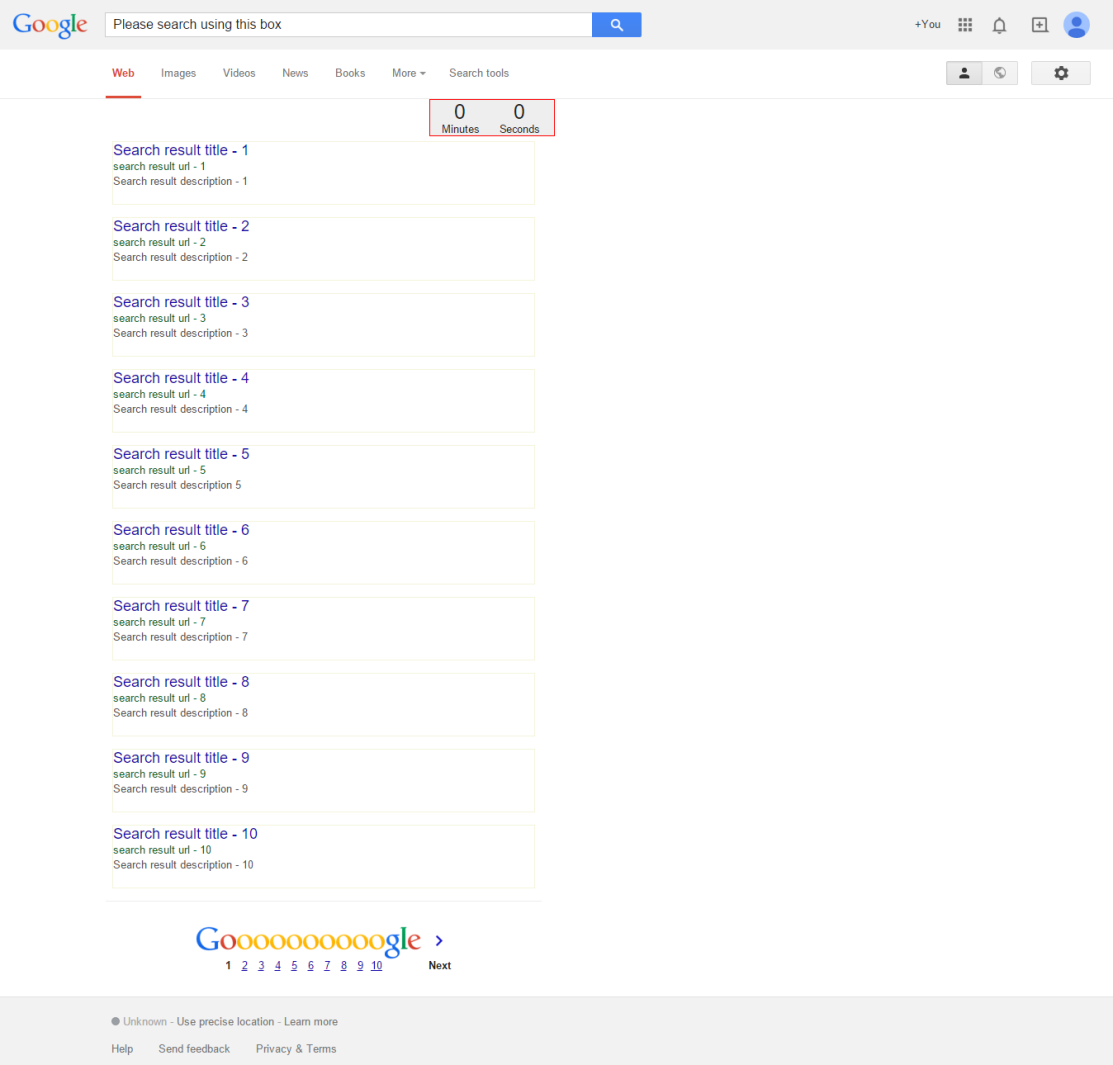
Search result template mimicking Google’s search used during the experiment.

**Figure 2:**
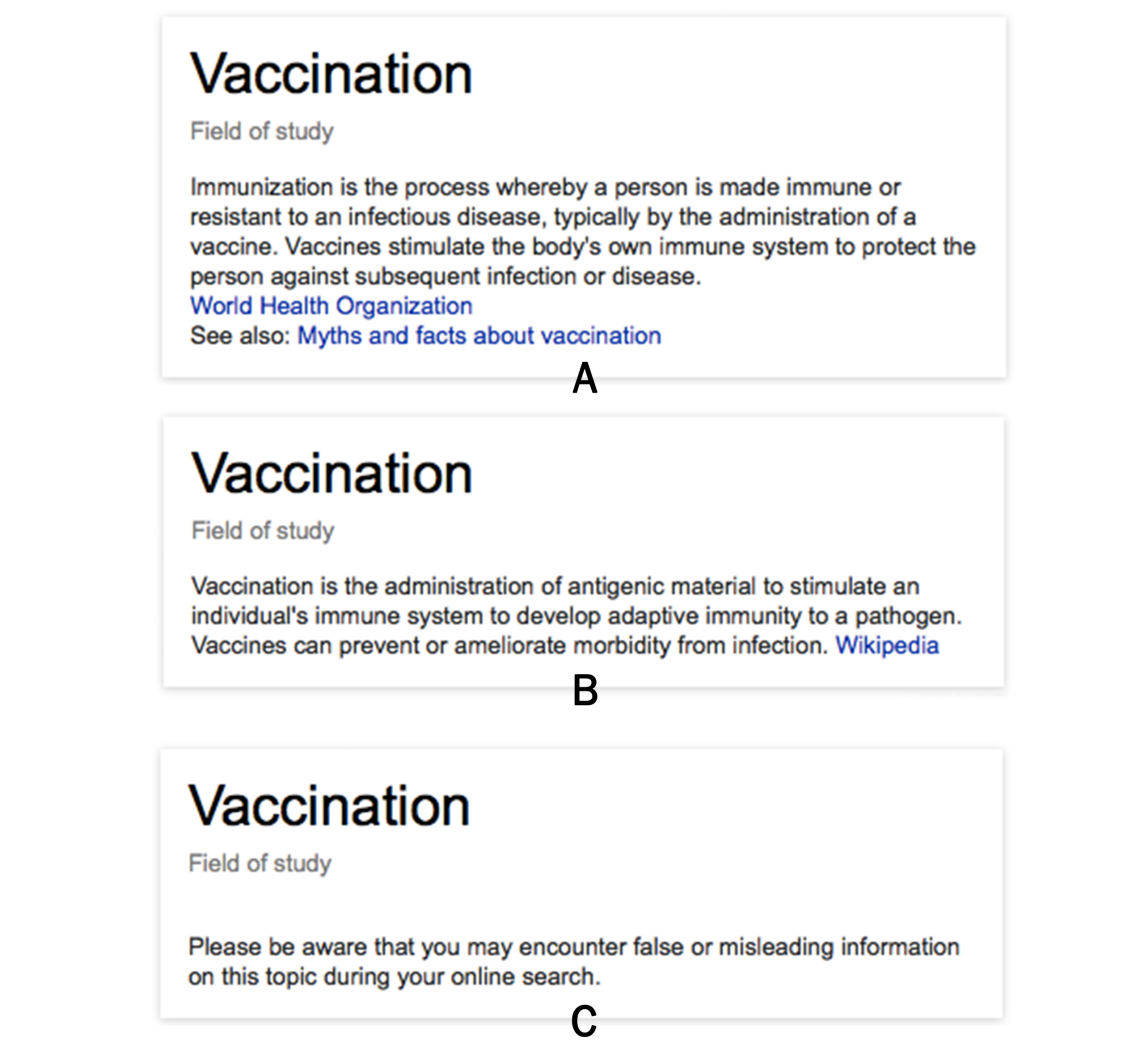
Three examples of the knowledge graph box as it appeared on the screen during the search: (A) Group 1 (comprehensible information from WHO), (B) Group 2 (hardly comprehensible information from Wikipedia), (C) Group 3 (warning message).

In addition to the manipulation of the knowledge graph box, all groups were exposed to the traditional search results as displayed in the Google search template (Figure 1). The knowledge graph box (displaying basic factual information and/or the warning) as well as the search results page implemented in this experiment used the same template (eg, design, fonts, colors) as provided by Google. Figure 2 depicts the template used showing the manipulated experimental factors (different versions of basic information and the warning message) as well as their corresponding experimental groups as mentioned in Table 1.

### Search Engine Manipulation

In all six groups, Google was manipulated to display a ratio of 50% pro- and 50% antivaccination websites as search results in a randomized order. This manipulation was realized by configuring the context and annotation files of the Google custom search engine [23,51]. In short, two custom search engines were built: one restricted to search from a set of pro-vaccination websites (ProVaccineSearcher) and the other to a set of antivaccination websites (ConVaccineSearcher). Thereby, the set of websites belonging to the ProVaccineSearcher contained only websites that had an overall positive standpoint toward vaccination, while the ConVaccineSearcher consisted of a set of webpages with an overall negative standpoint toward vaccination. All websites were content analyzed and subsequently categorized following the descriptions in [23,52]. By using JavaScript and the Google custom search application programming interface (API) [51], we controlled the search execution of both search engines and the display of the search results delivered from both [53]. Once a user entered a search query that matched the topic of the search task, the two engines were launched with the same query. The retrieved results were the first five relevant items (as chosen by the unmanipulated Google retrieval algorithm) from the pro-vaccination Web domains and similarly the first five items from the antivaccination Web domains. The retrieved results from both sources were combined and shuffled in a random order before being displayed. This was done for the construction of every search results page. Overall, there were 10 search results pages, with each page displaying 10 results, which mirrors Google’s default search template. The random shuffling guaranteed control of any order effects in the display of the pro- and antivaccination search results.

### Recruitment and Participants

A Web platform using the Drupal framework, which is an open source content management system [54], was developed and customized for the experiment. We designed a human intelligence task (HIT) asking potential participants from CrowdFlower [55] to go to the Web platform we prepared. Prior to the actual recruitment, we pretested the experiment in two consecutive rounds, again using CrowdFlower, to check for the functioning of the manipulation’s components, usability, and task understanding. After this pretest, we proceeded with the launching of the data collection. The recruitment advanced in sequential batches of on average 50 participants per HIT and lasted for 3 weeks in early 2015. Participants, registered as CrowdFlower workers, who passed the qualification requirements described subsequently were able to preview the HIT and apply for it. On completion of each batch, we analyzed the submissions and accepted people who followed the experiment as explained. There were two qualification requirements for the HIT: (1) users had to have the highest quality rating (Level 3, that is users who showed to be reliable and demonstrated high performance in completing posted tasks on the CrowdFlower platform), and (2) users needed to be located in an English-speaking country. The duration for task completion was set to 30 minutes.

### Experimental Procedure

Once the HIT was published on the CrowdFlower platform, qualified users were able to select it. The HIT forwarded the user to the Web platform that proceeded in several steps in multiple sliding screens. In the first screen, a short introduction to the experiment was presented. After giving informed consent and the CrowdFlower ID on the next page, participants were presented with a screen explaining the search task. Participants were randomly assigned to one of the five experimental or the control groups. All participants were asked to search for information about vaccination for 10 minutes and started their search on the manipulated version of Google. They were free to enter any kind of vaccination-related keyword that came to their mind. Examples of entered search queries are “vaccination,” “measles vaccine,” “flu vaccine,” or “vaccination pros and cons.” A word cloud displaying the entered search queries according to their frequency is displayed in Multimedia Appendix 2. If participants entered keywords that were not related to vaccination, they received a message reminding them of the relevant search topic. This was done in two ways: (1) by pre-processing the entered search queries and matching the entered keywords to a regular expression that included the various inflected forms of the vaccination concept including the misspelled ones and/or by (2) extracting the noun phrases and using the comprising keywords to query the freebase API [56] for determining the relevance of the search query to viral and infectious diseases. This allowed for matching vaccine names (eg, “mmr,” “measles,” or “chickenpox”). During the search, participants could enter as many search queries as they wished and open all search results they were interested in. Participants in the experimental groups received, in addition to the search results, a knowledge graph box that was displayed on the upper right-hand side of the screen. After 10 minutes, participants were guided to the questionnaire that took around 10-15 minutes to complete. Participants were debriefed through the CrowdFlower platform after the data collection had ended.

The platform was designed to capture users’ search behavior by recording their issued search queries, mouse hovers, clicks and selection of search elements, and their transition from one screen to another during the whole experiment. Video demos of the experimental workflow and manipulation can be found in Multimedia Appendix 3.

### Measures

#### Posttest Items

Participants were asked to fill in a posttest-questionnaire after the search. The questionnaire was pretested to avoid biased results due to language issues and contained the following measures used in the subsequent analyses:

1. Perceived utility of the knowledge graph box: semantic differential consisting of 6 items such as helpful/disturbing or comprehensible/ambiguous.

2. Perceived quality of the information in the knowledge graph box: Battery of 7 items asking, for example, whether the information in the knowledge graph box was considered relevant (0=highly irrelevant to 6=highly relevant), credible (0=I completely agree to 6=I completely disagree), or comprehensible (0=I completely agree to 6=I completely disagree), adapted from [23].

3. Evaluation of the websites: Battery of 10 items asking, for example, whether the information found on the websites was considered relevant (0=highly irrelevant to 6=highly relevant), credible (0=I completely agree to 6=I completely disagree), or comprehensible (0=I completely agree to 6=I completely disagree), adapted from [23].

4. Beliefs and attitudes toward vaccination: Set of 7 items consisting of statements about risks and benefits of vaccination that were answered on a Likert scale (1=completely disagree to 7=completely agree). Two further items measured the perceived likelihood of severe side effects after a child or an adult got vaccinated (0=very unlikely to 6=very likely), all adapted from [23].

5. Knowledge about vaccination: Validated scale consisting of 9 true/false items [57]. Knowledge scores were calculated for each participant.

6. Sociodemographic information: 9 items asking participants about, for example, their gender, age, educational level, nationality, vaccination status, profession, or perceived confidence in their information seeking skills.

Measures 1 and 2 were applied only in the experimental groups. The complete questionnaire is provided in Multimedia Appendix 4.

#### Outcome Variables

##### Knowledge About Vaccination

A knowledge score was computed for every participant by summing the scores of all items of the knowledge about vaccination measure [57].

##### Beliefs and Attitudes Toward Vaccination

An exploratory factor analysis was run on the posttest items measuring participants’ beliefs and attitudes toward vaccination and their evaluation of the search results with the goal to identify the latent variables, respective constructs. Given that some of the items were added or slightly modified from the ones used in [23], we aimed at finding the latent constructs using the responses from the current experiment and consequently obtaining factor scores using the items loaded on the emerging factors. Additionally, the emerging factors were validated by comparing them and their corresponding item loadings to the ones reported in [23].

#### Evaluation of the Knowledge Graph Box

##### Perceived Utility of the Knowledge Graph Box

The perceived utility of the knowledge graph box was assessed by computing a total sum score of the posttest items reported in Section 1. The item responses were coded from 0-6 and hence, the perceived utility score ranged from 0-36. The higher the score, the higher the perceived utility of the knowledge graph box.

##### Perceived Quality of the Information in the Knowledge Graph Box

The second measure was represented by the sum of the posttest items in Section 2, each measuring trust, correctness, persuasiveness, relevance, credibility, and comprehensibility of the information embedded in the knowledge graph box. Similar to the first evaluation measure (perceived utility), the responses were coded from 0-6. This measure represented the perceived quality of the knowledge graph box’s information with a total score ranging from 0-36. Again, a higher score indicated a higher perceived quality of the information.

### Data Analysis

Randomization check was completed to investigate if there were differences among the groups that should be taken into account in subsequent analyses. Results were insignificant for all items measuring sociodemographic variables, implying that there were no significant differences among the experimental groups (see Multimedia Appendix 5).

To test Hypotheses 1-4 and answer Research Question 1, two-way analyses of variance (ANOVAs) were conducted, one for each outcome variable with availability of basic information and the presence of a warning as independent variables.

Research Question 2 was answered by using two-way ANOVAs for the dependent variables perceived utility of the knowledge graph box and perceived quality of the information embedded in the knowledge graph box with the availability of basic information and the presence of a warning as independent variables.

Two-way ANOVAs were chosen as the procedure for analysis since we aimed at investigating the separate main effects as well as the interaction effect of both independent variables on the dependent variables.

Statistical analyses were considered significant at *P* <.05. Moreover, post-hoc analysis was conducted for the observed significant results, if applicable. Bonferroni correction and Tukey HSD were applied to control for the family-wise error such as increased probability of Type I error due to multiple comparisons. The analysis of self-reported measures was conducted using SPSS 20.

Additionally, we looked at heatmaps that we generated from the recorded search hovers to detect if hovering patterns over the knowledge graph box are different among the experimental groups.

## Results

The sample size was N=279 (group 1: n=45, group 2: n=45, group 3: n=46, group 4: n=50, group 5: n=47, group 6 [control group]: n=46). Over half (54.8%, 153/279) of participants were male and 45.2% (126/279) were female. The majority of participants came from the United States (43.4%, 121/279), followed by 29.4% (82/279) from the United Kingdom, 18.6% (52/279) from Canada, 1.8% (5/279) from Australia, and 1.1% (3/279) from New Zealand. The remaining respondents came from other countries. Just over half (52.0%, 145/279) of the participants were college graduates or had completed postgraduate studies, 22.2% (62/279) had concluded some college level, 5.7% (16/279) had received post-high school vocational or technical training, and 20.1% (56/279) had at least 8 years of high school or a completed high school degree. The mean age of participants was 37.34 years (SD 10.67), with 19 years as minimum and 69 years as maximum age.

### Knowledge About Vaccination

**Figure 3:**
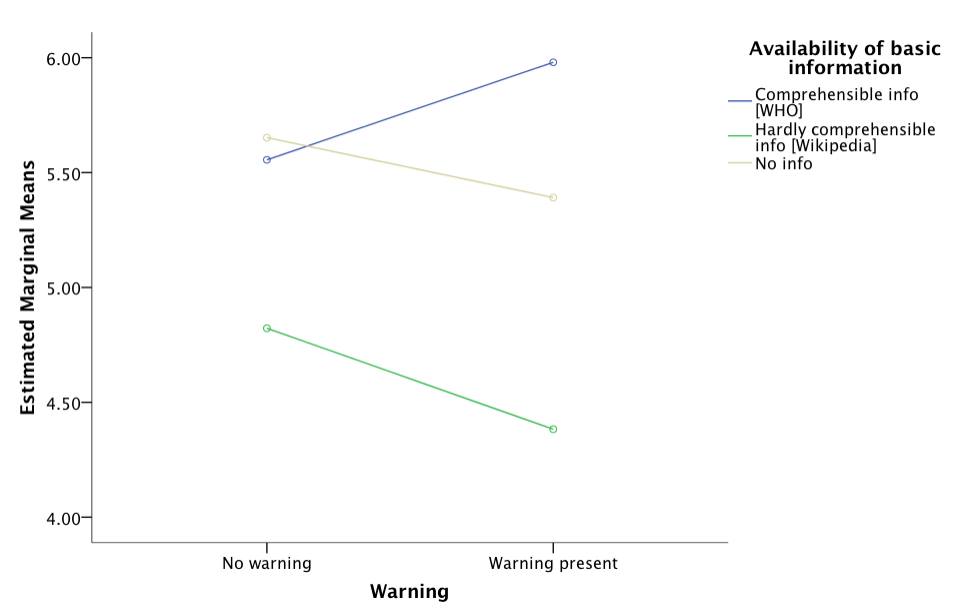
Posttest knowledge about vaccination as dependent on presence of a warning and comprehensibility level of basic information.

Using two-way ANOVA, there was a significant main effect of availability of basic information in the knowledge graph box on participants’ knowledge (*F*_2,273_=4.86, *P*=.01). Presence of the warning did not have a significant main effect on knowledge, nor was there an interaction effect. Following up with a post-hoc analysis with Bonferroni correction and Tukey HSD, participants belonging to Groups 1 and 4 receiving comprehensible information with or without the warning, combined, had significantly higher mean scores on the knowledge scale compared to the participants in Groups 2 and 5 who were exposed to the hardly comprehensible information (mean difference 1.18, *P*=.01). Figure 3 shows the estimated marginal means of the knowledge score by both experimental factors. It is worth noting that the warning increased knowledge in participants who received the comprehensible information while it decreased knowledge in those who received the hardly comprehensible information as well as in those who did not get information at all. Hence, Hypothesis 1 is partially supported while Hypothesis 3 is not.

### Beliefs and Attitudes Toward Vaccination

#### Factor Analysis

Factor analysis was run on 19 items (posttest items in Sections 3 and 4) with oblique rotation (promax) using the maximum likelihood extraction method. The Kaiser-Meyer-Olkin (KMO) measure verified the analysis’s sampling adequacy (KMO=.90). Bartlett’s test of sphericity (χ^2^_153_=3495.26, *P*=.001) indicated that correlations between items were sufficiently large for factor analysis. An initial analysis was run to obtain eigenvalues for each component in the data. Three eigenvalues were greater than Kaiser’s criterion of 1 and in combination explained 65% of the variance. Based on the parallel analysis and the scree plot in which an inflection point was detected, three factors were retained for final analysis. To assess the reliability of the subscales that emerged from factor analysis, we measured Cronbach's alpha. Table 2 shows the pattern matrix of the factor analysis including each item’s loadings on the obtained three factors. The eigenvalues were equal to 7.3, 3.9, and 1.1, and the percentage of variance explained by each was 38.4%, 20.7%, and 5.8%. In addition, Cronbach's alpha for each subscale comprising the items that showed loadings greater than or equal to 0.4 was .91, .89, and .89, respectively. The items forming factor 1 suggest a representation of skepticism or fear of vaccination side effects. Factor 2 displays an evaluation of information quality and factor 3 the acknowledgment of vaccination benefits. Factors 1 and 3 were negatively correlated (-.71), while both showed an insignificant positive correlation (.13, .05) with factor 2. The emerged factors and comprised items mirror the ones reported in [23].

**Table 2:** Exploratory factor analysis of beliefs and attitudes measures.

Pattern matrix^a^	Factor loadings		
	1	2	3
In your opinion, how likely is the occurrence of serious side effects after a child got vaccinated?	0.85^b^		
When I read about the possible side effects of vaccination on the websites, I felt worried.	0.82^b^		
When recommending vaccination, physicians do not pay enough attention to the possible side effects.	0.79^b^		
In your opinion, how likely is the occurrence of serious side effects after an adult got vaccinated?	0.77^b^		
When I read what the websites said about the effectiveness of vaccination, I felt worried.	0.76^b^		
Many vaccinations today do more harm than good.	0.75^b^		
Many of the vaccinations recommended today are redundant because the disease is almost extinct.	0.64^b^		
Vaccination often does not fully protect against a disease.	0.52^b^		
Thinking about the 10 minutes you spent searching for information on vaccination: Has the search made you more skeptical about vaccination or has the experience increased your confidence in it?	-0.38		
How convincing did you find the websites you looked at during your search?		0.91^b^	
The information on the websites I read was credible.		0.88^b^	
How much do you trust the information about vaccination you found on the websites before?		0.86^b^	
Was the information you found on the websites relevant?		0.73^b^	
How much do you trust Google to provide you with good information?		0.63^b^	
The information about vaccination I have just read on the websites was comprehensible for me.		0.52^b^	
If it weren’t for vaccination, many people would have a shorter lifespan today than they do.			0.86^b^
People who opt out of vaccination do not only put themselves at risk, but also other people.			0.74^b^
Vaccination is one of the greatest medical breakthroughs affecting our lives.			0.74^b^
In my opinion, people should follow the advice to get vaccinated.			0.60^b^

^a^Extraction method: maximum likelihood. Rotation method: Promax with Kaiser normalization.

^b^Loading ~ 0.4.

#### Factor 1: Skepticism/Fear of Vaccination Side Effects

Two-way ANOVA was used for investigating the effect of the two experimental factors (availability of basic information and warning message) on the scores of the first factor (skepticism/fear of vaccination side effects). The only detected significant effect was the main effect of the availability of basic information on the factor 1 scores (*F*_2,273_=3.5, *P*=.03).

**Figure 4:**
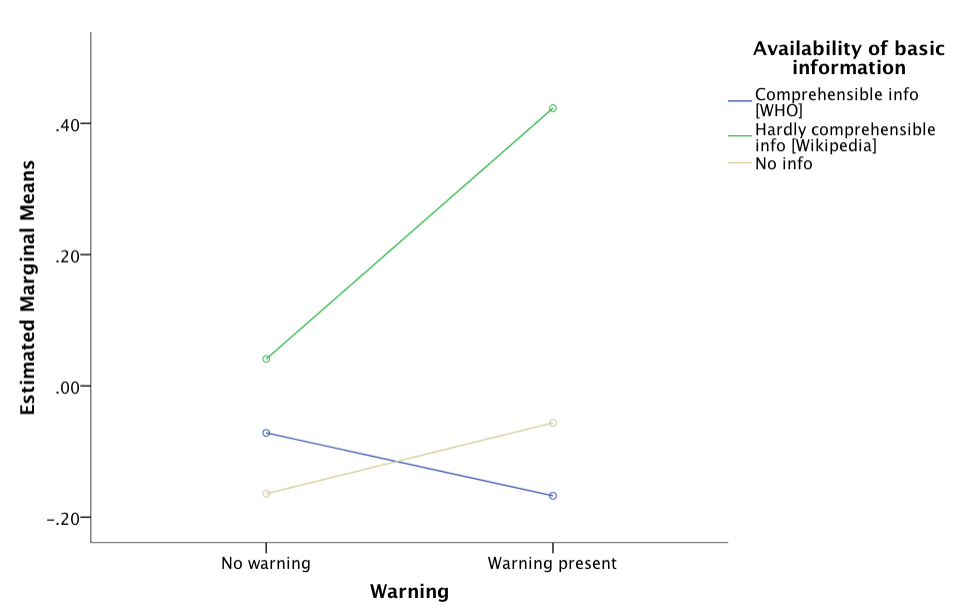
Skepticism/fear of vaccination side effects as dependent on presence of a warning and comprehensibility level of basic information.

Following up with a post-hoc analysis using Tukey HSD, participants receiving a knowledge graph box displaying comprehensible information (Groups 1 and 4) had significantly lower mean scores compared to the ones getting a knowledge graph box containing hardly comprehensible information (Groups 2 and 5; mean difference -0.36, *P*=.047). However, when using Bonferroni correction, the *P* value was equal to .05 at the threshold of rejecting the null hypothesis. This result is a consequence of the conservative nature of Bonferroni correction, which is used to control for Type I error by testing on a lower alpha-level that is, in our case, equal to 0.016 (eg, 0.05/3). Overall, participants exposed to comprehensible information with or without the warning were less afraid/skeptical of vaccination side effects compared to the ones receiving less comprehensible information, again regardless of the presence of a warning message. Figure 4 shows that the warning message reduces the skepticism among the group exposed to the comprehensible information. However, the opposite is true for the other groups.

#### Factor 2: Information Quality

**Figure 5:**
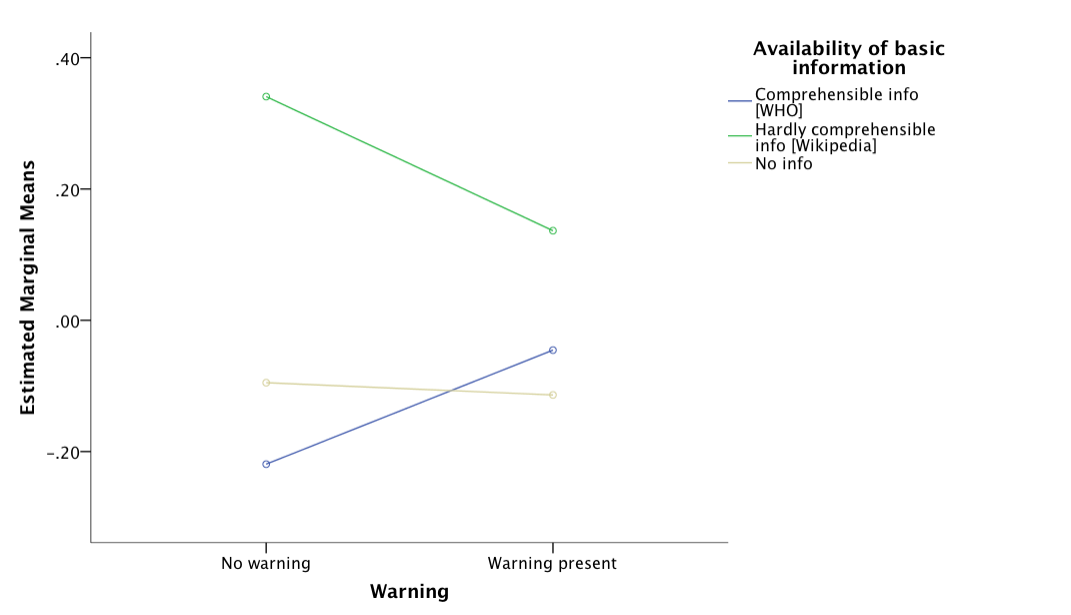
Attitude toward information quality as dependent on presence of a warning and comprehensibility level of basic information.

Using two-way ANOVA, the only detected significant effect was the main effect of availability of basic information on the scores of factor 2 (*F*_2,273_=3.73, *P*=.02). Following up with a post-hoc analysis, participants belonging to the groups receiving a knowledge graph box with comprehensible information (Groups 1 and 4) had significantly lower mean scores as compared to both groups getting a knowledge graph box displaying hardly comprehensible information (Groups 2 and 5; mean difference -0.36, *P*=.049 using Bonferroni correction and *P*=.043 using Tukey HSD). In other words, the former groups seem to be more critical in terms of the evaluation of information quality compared to the latter groups. Figure 5 shows the comparison between groups receiving the same type of basic information and the ones that additionally saw a warning message. Again, the warning appears to have a positive influence on the group receiving comprehensible information. The reverse pattern emerges for the group being exposed to hardly comprehensible information and slightly also for the group not getting basic information.

#### Factor 3: Acknowledgment of vaccination benefits

**Figure 6:**
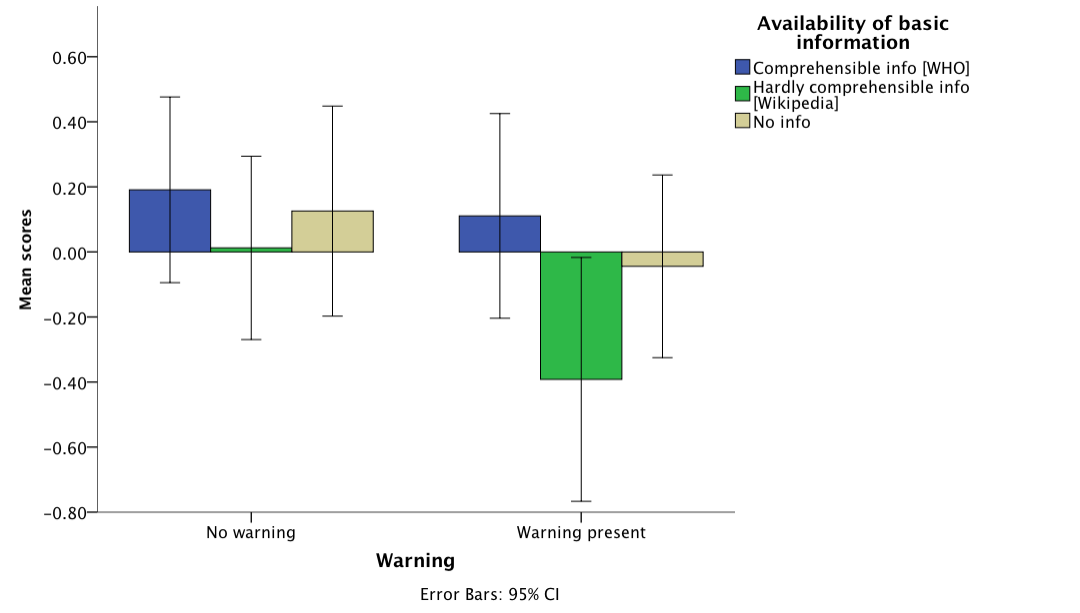
Acknowledgment of vaccination benefits as dependent on presence of a warning and comprehensibility level of basic information.

There was no significant effect with respect to the main effects of either experimental factor or for the interactions on the scores of Factor 3. However, groups receiving comprehensible basic information in the knowledge graph box, regardless of the presence of a warning, had the highest average scores on Factor 3 indicating a higher appreciation of vaccination benefits (see Figure 6).

Thus, Hypothesis 2 is partially supported while Hypothesis 4 is not. Moreover, there was no significant interaction (on all levels of experimental variables) between the availability of information and the warning on the outcome measures (RQ1). However, the overall results suggest that there was an interaction with regard to the groups receiving basic information (comprehensible and hardly comprehensible) and the corresponding groups with the added warning message.

### Evaluation of the Knowledge Graph Box

Two-way ANOVA was conducted for each dependent variable (perceived utility and perceived quality of the information displayed by the knowledge graph box) with the availability of basic information and the presence of a warning as independent variables. This time, five groups were compared since the control group was not exposed to the knowledge graph box and hence did not answer the items evaluating it.

In both cases, there was no significant effect with respect to the main effects of either experimental factor or of the interactions on the scores of the dependent variables. On average, the groups scored 24.4 out of 36 on perceived utility and 23.3 out of 36 on perceived information quality embedded in the knowledge graph box. This amounts to an average evaluation of 67.7% and 64.7%, suggesting an overall moderate to positive evaluation of the knowledge graph box. Heatmaps generated from the aggregated mouse hovers per group over the search elements showed similar hovering patterns among the groups (see Multimedia Appendix 6).

## Discussion

### Principal Findings

The findings suggest a positive effect of comprehensible basic information on information seekers’ vaccination-related knowledge and attitudes and a moderate to positive evaluation of the knowledge graph box among all groups. In contrast, the sole presentation of a warning message did not result in any substantial effects. Also, there was no significant interaction between the basic factual information and the warning.

The observation of a positive influence of easily comprehensible text is in line with previous research suggesting a higher persuasiveness of comprehensible information [44,45]. In this study, comprehensibility might have had a persuasive character in the sense of fostering participants’ agreement with considering vaccination as a form of health protection. The warning message, however, did not yield comparable effects. This could be explained by a lacking reference point for the information seekers. Literature on warning messages came to the conclusion that a warning is most successful when it is combined with advice for action such as a strategy to avoid the threat [48]. Thus, the warning message by itself might have disrupted consumers’ biased search but since a strategy stating how to handle or detect misleading information was missing, they could not translate this knowledge about the presence of false information into more successful search outcomes.

Interestingly, the combination of comprehensible information and a warning message supports this assumption, although on a non-significant level. That is, the obtained results suggest that comprehensible information combined with a warning steers consumers toward a vaccination-supporting position. Since the comprehensible information might have been interpreted by the participants as promoting vaccination, the alert could have been linked to the information and consequently been perceived as warning against antivaccination information. In contrast, the combination of hardly comprehensible information and a warning seems to direct information seekers more toward a vaccination-skeptical position. This result might be, on the one hand, due to the content of the Wikipedia article that was linked to the knowledge graph box. When clicking on it, participants did not only see information about the importance of vaccination and factual information (eg, history, functioning mechanisms, statistics) but also paragraphs related to side effects, adverse events, and the surrounding controversy. This might have sparked concerns about vaccination. On the other hand, for laypersons, the hardly understandable definition might have evoked confusion and insecurity among the health information seekers. In combination with a warning lacking polarity or a clear positioning, these doubts might have been augmented, leading to the opposite effect of the warning as compared to the group receiving comprehensible basic information. A follow-up on the true relationship between these two experimental factors would be an interesting starting point for future research in this area.

The comparison of the results to the ones of a recent study investigating the effects of different ratios of pro- and antivaccination information displayed by Google [23] stresses the potential of this intervention in terms of effectiveness and applicability. When looking at the achieved factor scores, the groups exposed to comprehensible information in this experiment achieved better outcomes than the groups being exposed to an almost equal amount of pro- and antivaccination information in [23] as they were more knowledgeable, less skeptical of vaccination, and more critical of information quality. This is promising because it shows that filtering the retrieved search results according to their quality and source credibility is not the only approach to minimize the negative effects of a biased information search. Indeed, also the incorporation of comprehensible semantic information of the health-related search topic in the knowledge graph box appears to be successful, while being more feasible.

### Limitations

Although this study offers valuable insights into a new technological debiasing technique, some limitations need to be considered. First, we employed a design that immediately tested the effectiveness of a knowledge graph box as a debiasing technique without a prior phase that only observed the occurrence of a biased search and information processing. Acknowledging the solid body of research that demonstrates the deficiency of people’s information search behavior and processing [23,24,28,33-35], we took the problem context for granted, rendering another demonstration of the same phenomenon unnecessary.

A second limitation refers to the recruitment of participants through CrowdFlower. Although the sample was sufficiently diverse concerning sociodemographic characteristics such as age, gender, and nationality, a disproportionate share of participants was highly educated. However, Internet samples are overall more heterogeneous than traditional samples [58]. Yet, the probably higher level of online search experience and computer skills remains a limitation to the generalization of the findings. Using an online sample further bears the peril of “non-serious” or “repeat responders” [58]. Still, a biasing effect of such participants on the results of the study was minimized through a careful investigation and follow-up of each respondent. This was done in two ways. First, the developed platform captured participants’ interaction and behavior throughout the course of the experiment. This information was then used to construct a timeline for every participant to verify that the experiment was conducted in one sitting. Second, the respondents were identified during the experiment based on their inserted CrowdFlower ID. This allowed for a validation of the recorded data for every participant in our platform against the task-submission data on the CrowdFlower platform.

Moreover, the template Google currently uses when displaying the information in the knowledge graph box includes both the basic information and its source as a hyperlink. As a result, we cannot establish or compare a difference in contribution of the source and the information itself to the observed effect. Our emphasis on the importance of the information itself is, however, in line with the communication science-based assumption that the content of a message is more crucial than variables such as source credibility or attractiveness [43]. This assumption is also empirically supported by the equally high evaluation scores awarded to the knowledge graph box by the experimental groups, regardless of the displayed source. Nevertheless, this was only a first test of the possible effects of the knowledge graph box focusing on the differentiation between its overall presence and two content-related factors, namely comprehensibility of basic information and presence of a warning. Future research could experimentally distinguish between single components of the knowledge graph box and investigate a potential influence of the content’s source more thoroughly.

Eventually, we could have employed a pre-posttest design instead of a posttest-only design. Using a different experimental design would have allowed for assessing participants’ vaccination-related baseline knowledge and attitudes. However, we refrained from this alternative in order to not sensitize our participants to the experiment’s true outcome of interest, which could have had a biasing effect. Further, we aimed at comparing the findings to the studies reported in [23] by keeping the experimental design constant. Still, further research investigating the online health information seeking process by applying different experimental designs is needed.

### Conclusions

This study assessed the debiasing effects of a knowledge graph box providing health information seekers with basic information (comprehensible vs hardly comprehensible) and/or a warning message during their search on vaccination via Google. We intended to ensure a realistic setting of the experiment so that it could be implemented in real-life. That means we adhered to the technology currently used by Google to retrieve the semantic information for the knowledge graph box and used neutral information to design the experimental stimuli.

Since Google already uses the knowledge graph box [42], a simple change in the retrieval procedure for its content could make a valuable difference for the consumers. In fact, while this experiment was in the field, Google announced an update regarding the information displayed in the knowledge graph box when it comes to searching for health-related information [59]. The update concerns the retrieval of semantic information from “high-quality” websites such as the Mayo Clinic, WebMD, or Centers for Disease Control and Prevention [59]. However, this change pertained only to the United States and searches conducted in English [59]. Surprisingly, the update did not affect searches conducted on vaccination- or vaccine-related search terms at the time of writing this manuscript (see Multimedia Appendix 1).

Hence, the current situation suggests that there is still an extensive potential to improve the online health information seeking process, as well as a necessity for further research in this area. Instead of developing and studying complex interventions to improve people’s health information search, we suggest the further investigation of more realizable strategies like the one presented here.

## Appendix

Multimedia Appendix 1 Video demo of actual search on Google about vaccination (February 2016).

Multimedia Appendix 2 Word cloud of entered search queries.

Multimedia Appendix 3 Video demo of experimental groups.

Multimedia Appendix 4 Complete questionnaire.

Multimedia Appendix 5 Results of the randomization check.

Multimedia Appendix 6 Heatmaps showing the aggregated mouse hovering.

## Abbreviations

ANOVA: analysis of variance
API: application programming interface
HIT: human intelligence task
KMO: Kaiser-Meyer-Olkin
WHO: World Health Organization
