# Supplementary figures and images for: Manipulating Google’s Knowledge Graph Box to Counter Biased Information Processing During an Online Search on Vaccination: Application of a Technological Debiasing Strategy
Source: J Med Internet Res. 2016 Jun 2;18(6):e137. doi: 10.2196/jmir.5430 (PMC4911515; doi:10.2196/jmir.5430)

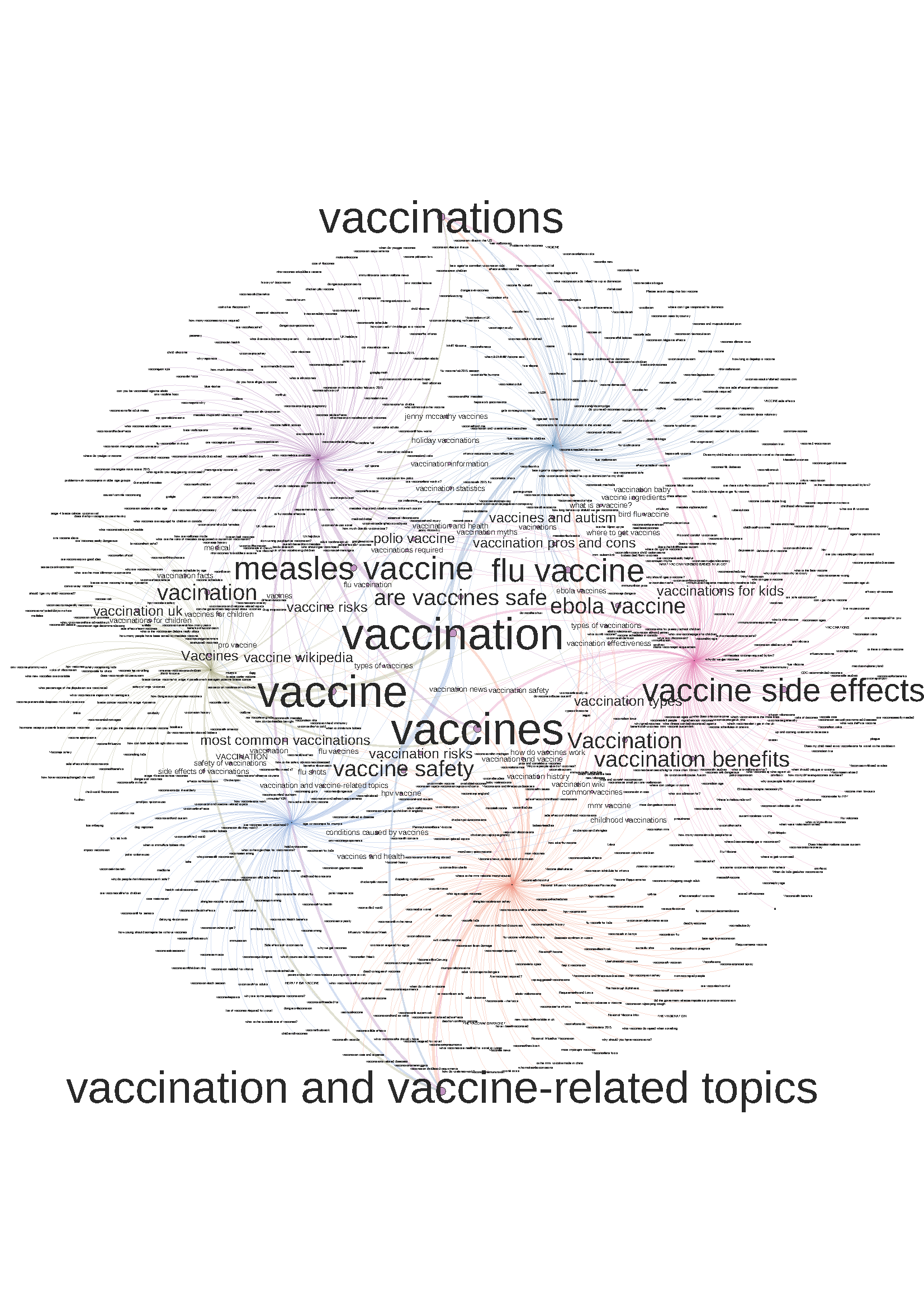

Supplement: Multimedia Appendix 2 [file jmir_v18i6e137_app2.png]
