# Supplementary material for: Manipulating Google’s Knowledge Graph Box to Counter Biased Information Processing During an Online Search on Vaccination: Application of a Technological Debiasing Strategy
Source: J Med Internet Res. 2016 Jun 2;18(6):e137. doi: 10.2196/jmir.5430 (PMC4911515; doi:10.2196/jmir.5430)
Supplement: Multimedia Appendix 4 [file jmir_v18i6e137_app4.pdf]

Manipulating Google's knowledge graph box to counter biased information processing during an online search on vaccination. Application of a technological debiasing strategy.

Screenshots of the questionnaire

Questionnaire, page 1: Media usage (Allam, Schulz, & Nakamoto, 2014)

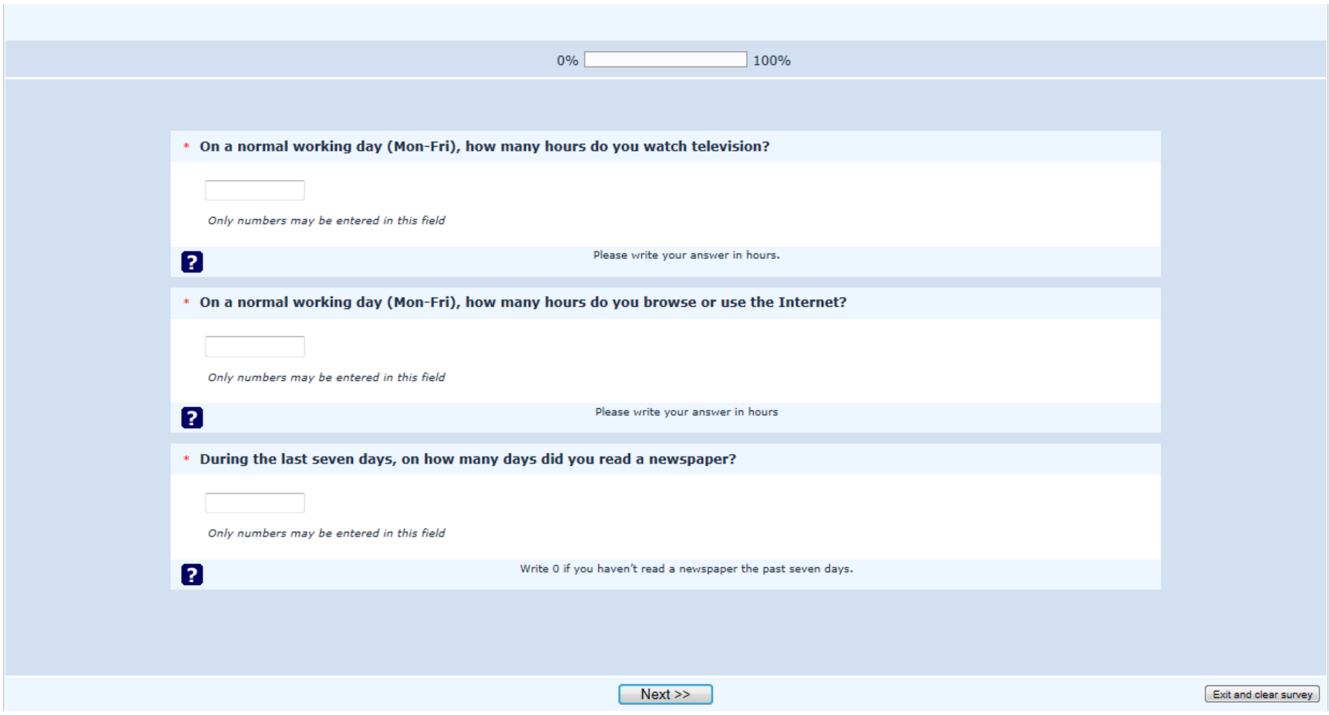
0% 100%

* **On a normal working day (Mon-Fri), how many hours do you watch television?**

Only numbers may be entered in this field

? Please write your answer in hours.

* **On a normal working day (Mon-Fri), how many hours do you browse or use the Internet?**

Only numbers may be entered in this field

? Please write your answer in hours

* **During the last seven days, on how many days did you read a newspaper?**

Only numbers may be entered in this field

? Write 0 if you haven't read a newspaper the past seven days.

Next >> Exit and clear survey

Questionnaire, page 2: Transition text

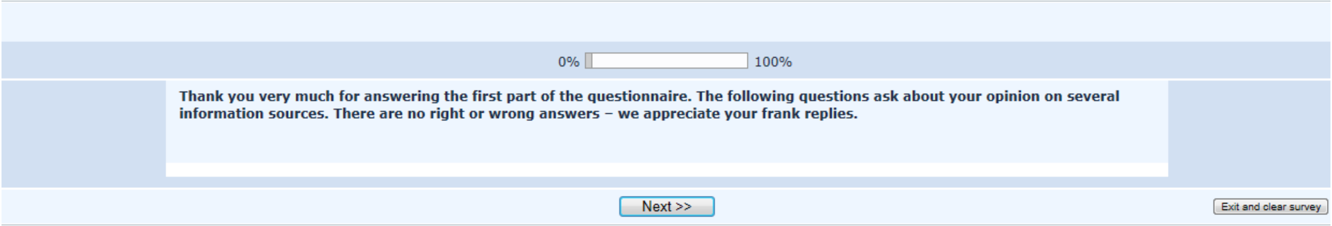
0% 100%

Thank you very much for answering the first part of the questionnaire. The following questions ask about your opinion on several information sources. There are no right or wrong answers – we appreciate your frank replies.

Next >> Exit and clear survey

Questionnaire, page 3: Perceived trustworthiness of information sources (HINTS, 2007 for general sources; self-developed questions for specific sources)

0% 100%

* In general, how much would you trust health information obtained from each of the following sources?

	A lot	Some	A litle	Not at all
Radio	○	○	○	○
Government health agencies	○	○	○	○
Television	○	○	○	○
Charitable organizations	○	○	○	○
Family or friends	○	○	○	○
Religious organizations and leaders	○	○	○	○
Newspapers or magazines	○	○	○	○
A doctor	○	○	○	○
The Internet	○	○	○	○

* In terms of specific sources, how much would you trust information about health or medical topics obtained from each of the following sources?

	A lot	Some	A little	Not at all	Don't know
Wikipedia	○	○	○	○	○
World Health Organization	○	○	○	○	○
Web MD	○	○	○	○	○
Centers for Disease Control and Prevention	○	○	○	○	○
Google News	○	○	○	○	○
Health Line	○	○	○	○	○
Health Ministry	○	○	○	○	○
National Library of Medicine	○	○	○	○	○

Next >> Exit and clear survey

Questionnaire, page 4: Transition text

0% 100%

In the following we would like to ask you some questions about the search on information about vaccination you just performed in the previous phase of this study.

Next >> Exit and clear survey

Questionnaire, page 5: Vaccination thought listing

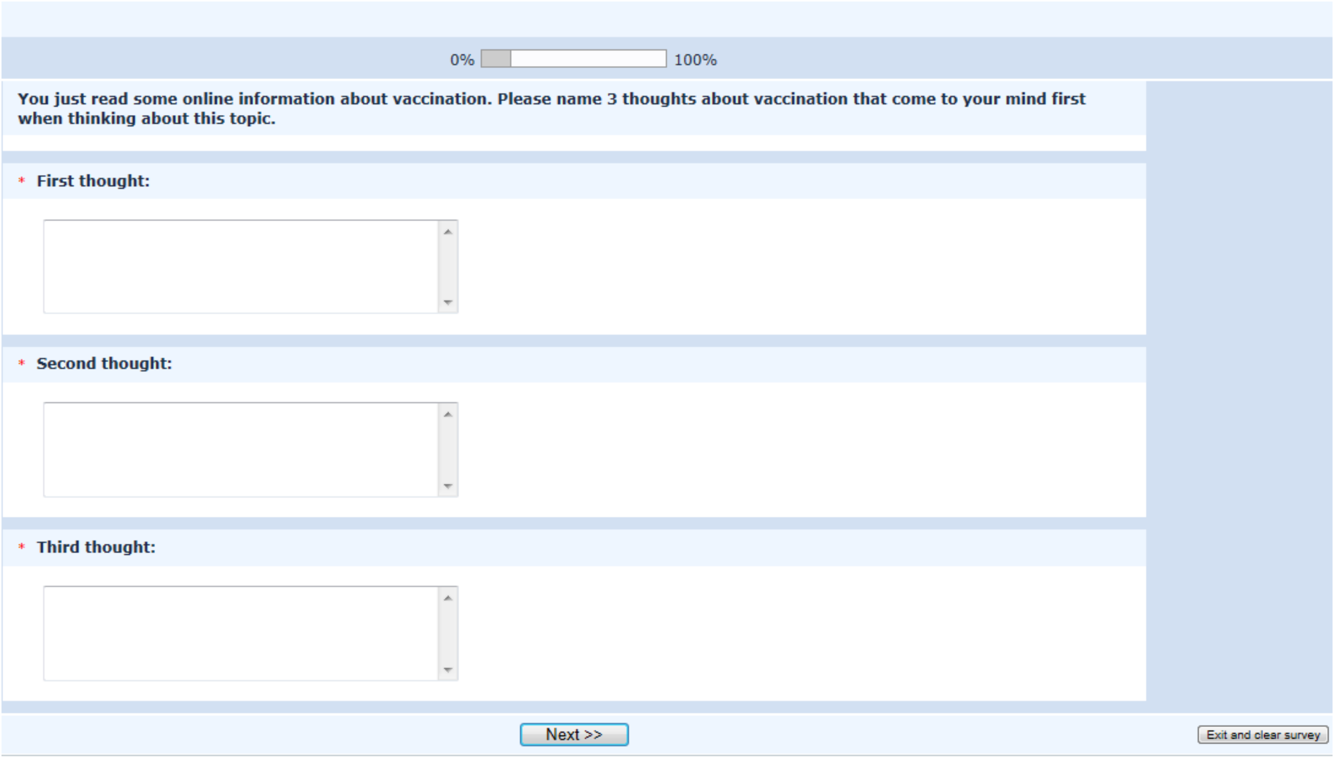
0% 100%

You just read some online information about vaccination. Please name 3 thoughts about vaccination that come to your mind first when thinking about this topic.

* First thought:

* Second thought:

* Third thought:

Next >>

Exit and clear survey

Questionnaire, page 6: Manipulation check I

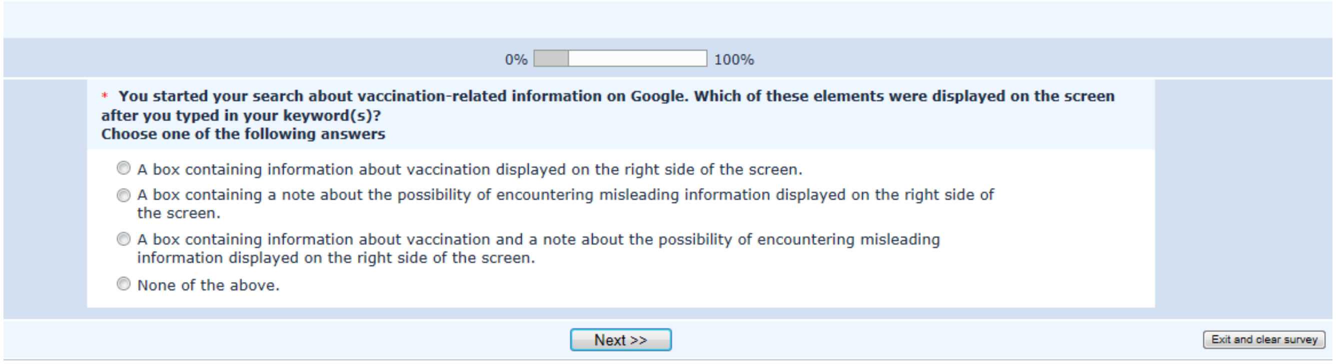
0% 100%

* You started your search about vaccination-related information on Google. Which of these elements were displayed on the screen after you typed in your keyword(s)?
Choose one of the following answers

- A box containing information about vaccination displayed on the right side of the screen.
- A box containing a note about the possibility of encountering misleading information displayed on the right side of the screen.
- A box containing information about vaccination and a note about the possibility of encountering misleading information displayed on the right side of the screen.
- None of the above.

Next >>

Exit and clear survey

Questionnaire, page 7: Manipulation check II

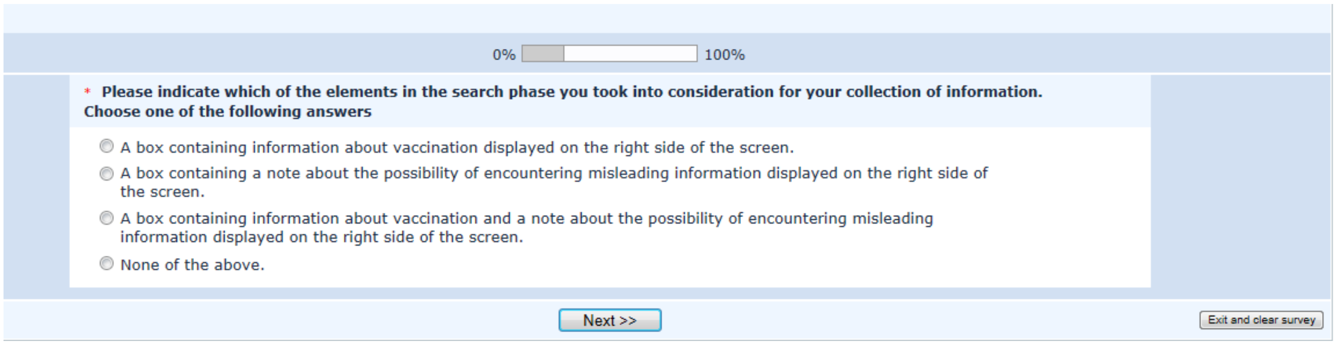

0% 100%

* **Please indicate which of the elements in the search phase you took into consideration for your collection of information.**
Choose one of the following answers

- A box containing information about vaccination displayed on the right side of the screen.
- A box containing a note about the possibility of encountering misleading information displayed on the right side of the screen.
- A box containing information about vaccination and a note about the possibility of encountering misleading information displayed on the right side of the screen.
- None of the above.

Next >>

Exit and clear survey

Questionnaire, page 8: Transition text for experimental groups

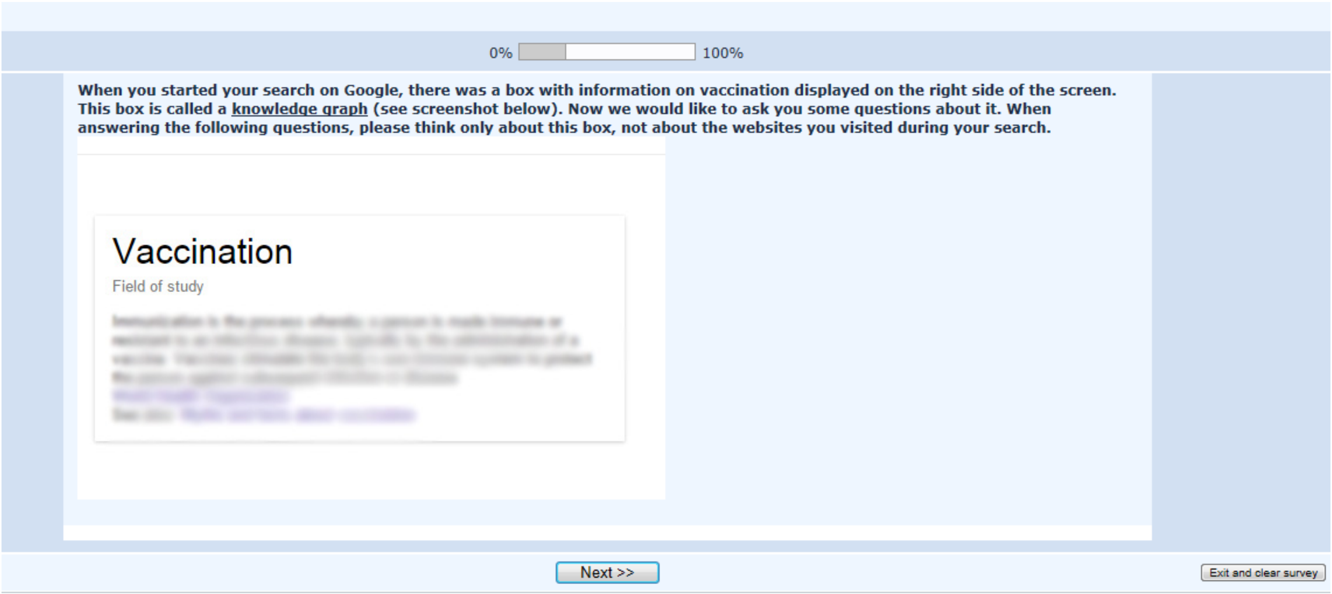

0% 100%

When you started your search on Google, there was a box with information on vaccination displayed on the right side of the screen. This box is called a knowledge graph (see screenshot below). Now we would like to ask you some questions about it. When answering the following questions, please think only about this box, not about the websites you visited during your search.

Vaccination

Field of study

Next >>

Exit and clear survey

Questionnaire, page 9: Perception of knowledge graph

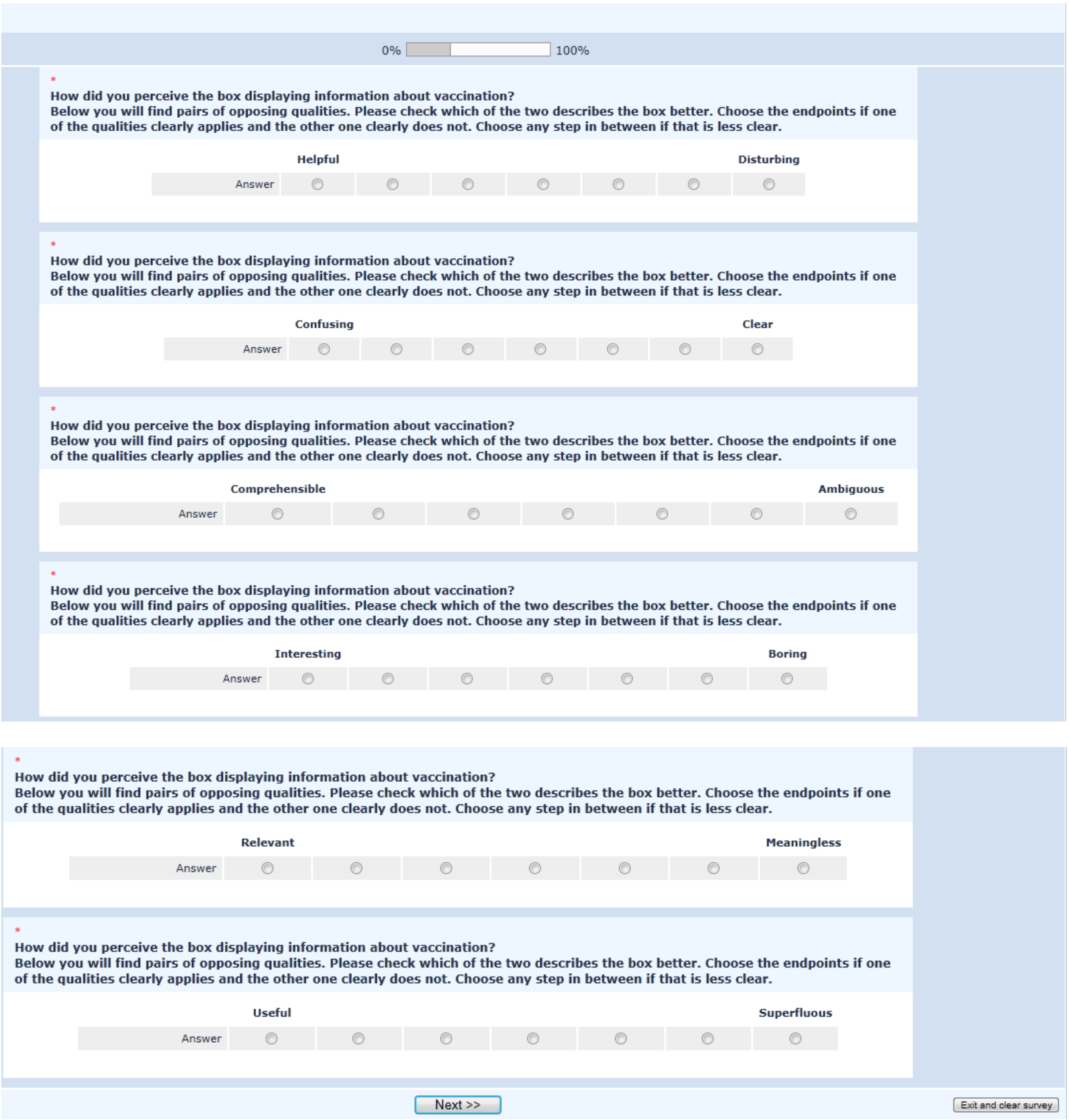
0% 100%

*
How did you perceive the box displaying information about vaccination?
Below you will find pairs of opposing qualities. Please check which of the two describes the box better. Choose the endpoints if one of the qualities clearly applies and the other one clearly does not. Choose any step in between if that is less clear.

Helpful — Disturbing

Answer ○ ○ ○ ○ ○ ○ ○

*
How did you perceive the box displaying information about vaccination?
Below you will find pairs of opposing qualities. Please check which of the two describes the box better. Choose the endpoints if one of the qualities clearly applies and the other one clearly does not. Choose any step in between if that is less clear.

Confusing — Clear

Answer ○ ○ ○ ○ ○ ○ ○

*
How did you perceive the box displaying information about vaccination?
Below you will find pairs of opposing qualities. Please check which of the two describes the box better. Choose the endpoints if one of the qualities clearly applies and the other one clearly does not. Choose any step in between if that is less clear.

Comprehensible — Ambiguous

Answer ○ ○ ○ ○ ○ ○ ○

*
How did you perceive the box displaying information about vaccination?
Below you will find pairs of opposing qualities. Please check which of the two describes the box better. Choose the endpoints if one of the qualities clearly applies and the other one clearly does not. Choose any step in between if that is less clear.

Interesting — Boring

Answer ○ ○ ○ ○ ○ ○ ○

*
How did you perceive the box displaying information about vaccination?
Below you will find pairs of opposing qualities. Please check which of the two describes the box better. Choose the endpoints if one of the qualities clearly applies and the other one clearly does not. Choose any step in between if that is less clear.

Relevant — Meaningless

Answer ○ ○ ○ ○ ○ ○ ○

*
How did you perceive the box displaying information about vaccination?
Below you will find pairs of opposing qualities. Please check which of the two describes the box better. Choose the endpoints if one of the qualities clearly applies and the other one clearly does not. Choose any step in between if that is less clear.

Useful — Superfluous

Answer ○ ○ ○ ○ ○ ○ ○

Next >>

Exit and clear survey

Questionnaire, page 10: Transition text

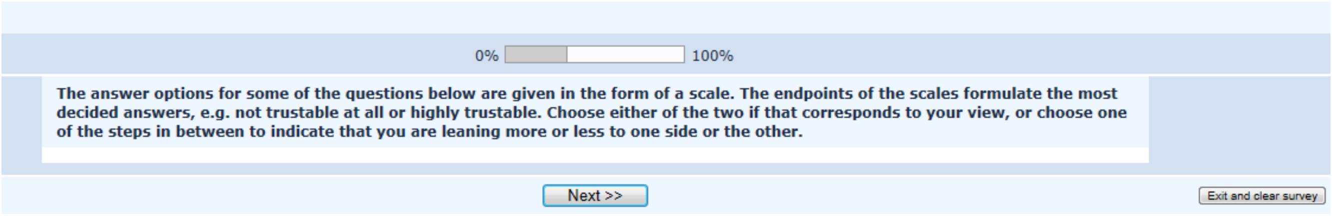
0% 100%

The answer options for some of the questions below are given in the form of a scale. The endpoints of the scales formulate the most decided answers, e.g. not trustable at all or highly trustable. Choose either of the two if that corresponds to your view, or choose one of the steps in between to indicate that you are leaning more or less to one side or the other.

Next >>

Exit and clear survey

Questionnaire, page 11: Evaluation of knowledge graph I (Allam et al., 2014)

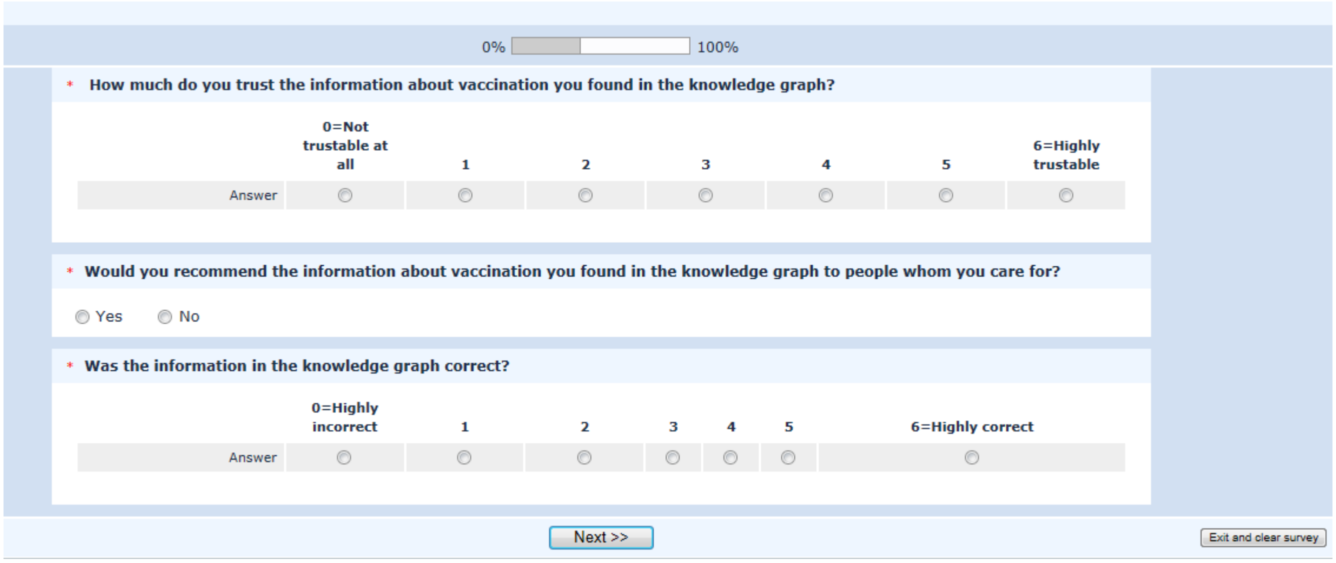
0% 100%

* How much do you trust the information about vaccination you found in the knowledge graph?

	0=Not trustable at all	1	2	3	4	5	6=Highly trustable
Answer	◯	◯	◯	◯	◯	◯	◯

* Would you recommend the information about vaccination you found in the knowledge graph to people whom you care for?

◯ Yes ◯ No

* Was the information in the knowledge graph correct?

	0=Highly incorrect	1	2	3	4	5	6=Highly correct
Answer	◯	◯	◯	◯	◯	◯	◯

Next >>

Exit and clear survey

Questionnaire, page 12: Evaluation of knowledge graph II (Allam et al., 2014)

0% 100%

* How convincing did you find the information that was displayed in the knowledge graph?

	0=Not convincing at all	1	2	3	4	5	6=Very convincing
Answer	○	○	○	○	○	○	○

* Was the information displayed in the knowledge graph relevant for you?

	0=Highly irrelevant	1	2	3	4	5	6=Highly relevant
Answer	○	○	○	○	○	○	○

* The information displayed in the knowledge graph was credible.

	0=I completely disagree	1	2	3	4	5	6=I completely agree
Answer	○	○	○	○	○	○	○

Next >>

Exit and clear survey

Questionnaire, page 13: Evaluation of knowledge graph III (Allam et al., 2014)

0% 100%

* The information displayed in the knowledge graph was comprehensible for me.

	0=I completely disagree	1	2	3	4	5	6=I completely agree
Answer	○	○	○	○	○	○	○

*
What do you think, where did the box come from?
I thought it was ..

Choose one of the following answers

- .. a service provided by Google.
- I did not think about that at all.
- .. a service provided by my web provider.
- .. paid for advertising space.
- .. a service provided by some official health promotion institution.

Next >>

Exit and clear survey

Questionnaire, page 14: Transition text for website evaluation

0% 100%

The following questions refer exclusively to the websites you found during your search on vaccination. So when answering, please consider only the information from the websites you read.
Again, the answer options for some of the questions are given in the form of a scale. The endpoints of the scales formulate the most explicit answers. Choose either of the two if that corresponds to your view, or choose one of the steps in between to indicate that you are leaning more or less to one side or the other.

Next >> Exit and clear survey

Questionnaire, page 15: Evaluation of websites I (Allam et al., 2014)

0% 100%

* **Do you have any second thoughts about vaccination now, after the search, because you are worried about possible side-effects?**
Choose one of the following answers

- Yes
- No
- Do not know

* **How much do you trust the information about vaccination you found on the websites before?**

	0=Not trustable at all	1	2	3	4	5	6=Highly trustable
Answer	○	○	○	○	○	○	○

* **How convincing did you find the websites you looked at during your search?**

	0=Not convincing at all	1	2	3	4	5	6=Very convincing
Answer	○	○	○	○	○	○	○

* **Was the information you found on the websites relevant?**

	0=Highly irrelevant	1	2	3	4	5	6=Highly relevant
Answer	○	○	○	○	○	○	○

Next >> Exit and clear survey

Questionnaire, page 16 : Evaluation of websites II (Allam et al., 2014)

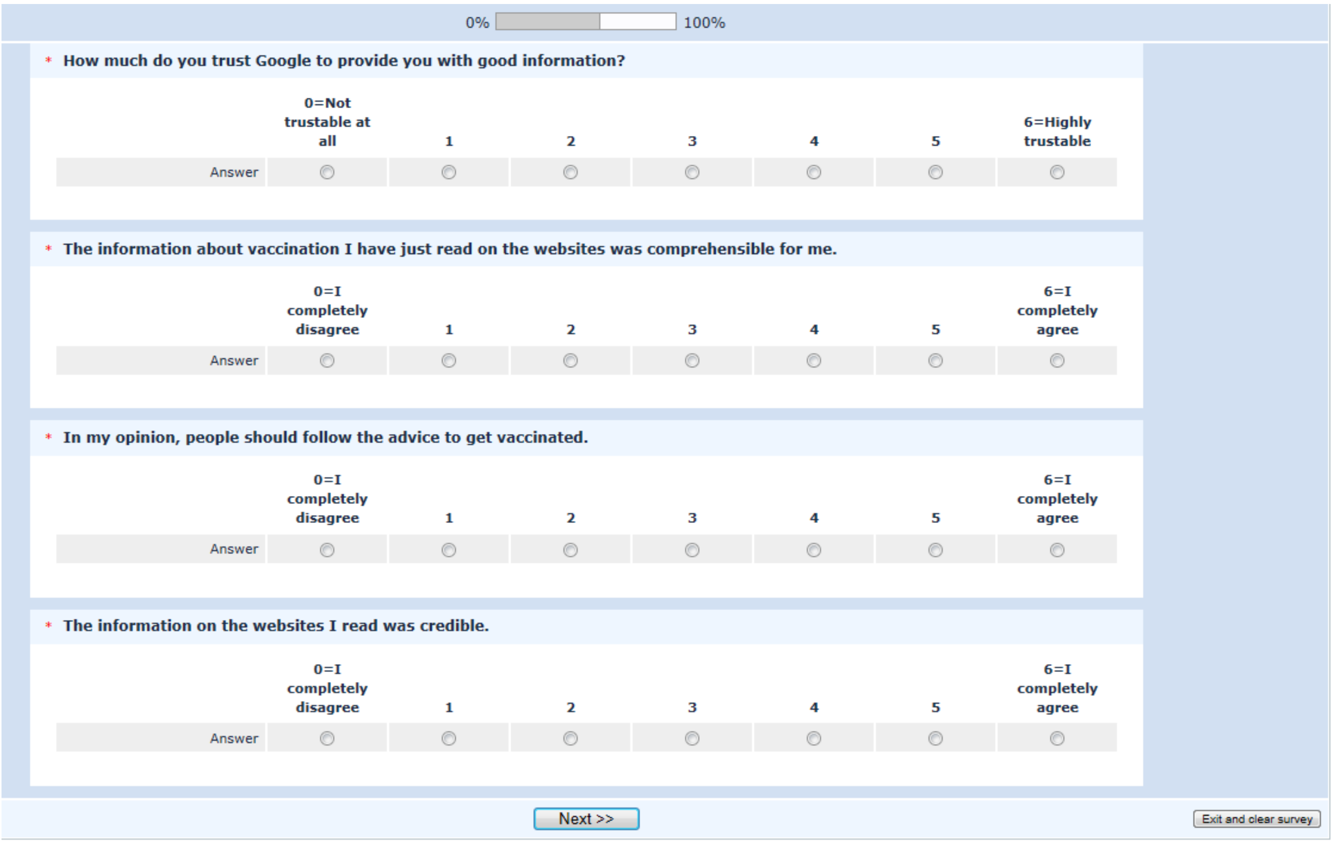

Questionnaire, page 17 : Evaluation of websites III (Allam et al., 2014)

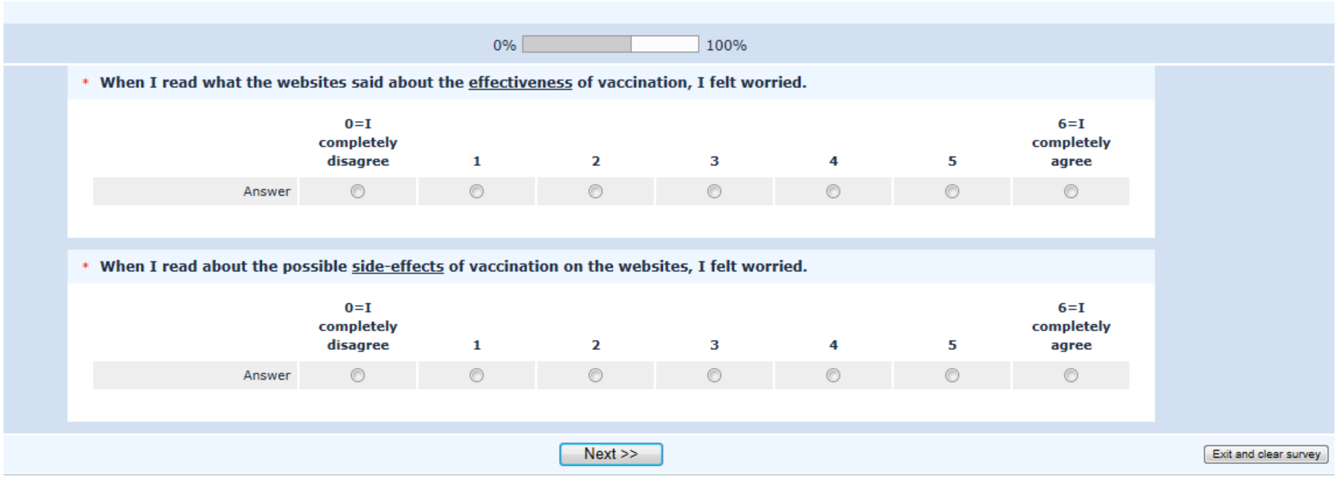

Questionnaire, page 18 : Transition text – general questions on vaccination

0% 100%

Again, we thank you very much for your answers so far. The next part of the questionnaire is about vaccination in general. Please read the questions carefully.

Next >> Exit and clear survey

Questionnaire, page 19 : Likelihood of side effects

0% 100%

* **In your opinion, how likely is the occurrence of serious side effects after a child got vaccinated?**

	0=Very unlikely	1	2	3	4	5	6=Very likely
Answer	○	○	○	○	○	○	○

* **In your opinion, how likely is the occurrence of serious side effects after an adult got vaccinated?**

	0=Very unlikely	1	2	3	4	5	6=Very likely
Answer	○	○	○	○	○	○	○

Next >> Exit and clear survey

Questionnaire, page 20 : Attitude toward vaccination (Allam et al., 2014)

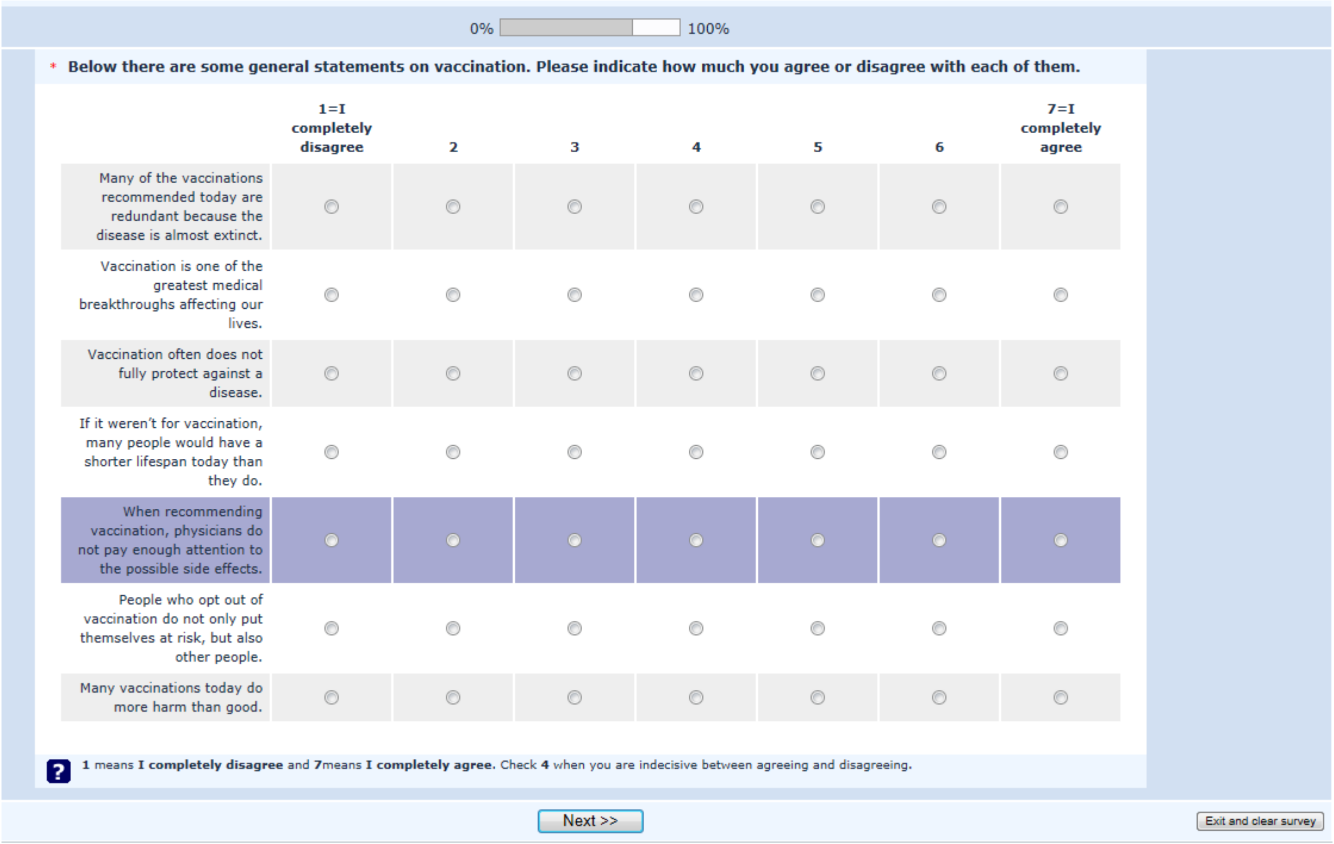

0% 100%

* Below there are some general statements on vaccination. Please indicate how much you agree or disagree with each of them.

	1=I completely disagree	2	3	4	5	6	7=I completely agree
Many of the vaccinations recommended today are redundant because the disease is almost extinct.	○	○	○	○	○	○	○
Vaccination is one of the greatest medical breakthroughs affecting our lives.	○	○	○	○	○	○	○
Vaccination often does not fully protect against a disease.	○	○	○	○	○	○	○
If it weren't for vaccination, many people would have a shorter lifespan today than they do.	○	○	○	○	○	○	○
When recommending vaccination, physicians do not pay enough attention to the possible side effects.	○	○	○	○	○	○	○
People who opt out of vaccination do not only put themselves at risk, but also other people.	○	○	○	○	○	○	○
Many vaccinations today do more harm than good.	○	○	○	○	○	○	○

? 1 means **I completely disagree** and **7**means **I completely agree**. Check **4** when you are indecisive between agreeing and disagreeing.

Next >>

Exit and clear survey

Questionnaire, page 21 : Vaccination knowledge scale (Zingg & Siegrist, 2012)

0% 100%

* **Please indicate whether the following statements are true or false, considering all you have ever heard about it.**

	True	False	Do not know
Vaccines are superfluous, as diseases can be treated (e.g. with antibiotics).	○	○	○
Diseases like autism, multiple sclerosis, and diabetes might be triggered through vaccinations.	○	○	○
Many vaccinations are administered too early, so that the body's own immune system has no possibility to develop.	○	○	○
Without broadly applied vaccine programs, smallpox would still exist.	○	○	○
The doses of the chemicals used in vaccines are not dangerous for humans.	○	○	○
Children would be more resistant if they were not always vaccinated against all diseases.	○	○	○
The efficacy of vaccines has been proven.	○	○	○
The immune system of children is not overloaded through many vaccinations.	○	○	○
Vaccinations increase the occurrence of allergies.	○	○	○

Next >> Exit and clear survey

Questionnaire, page 22 : Perceived skepticism after search

0% 100%

*

Thinking about the 10 minutes you spent searching for information on vaccination: Has the search made you more skeptical about vaccination or has the experience increased your confidence in it?

Choose one of the following answers

- ○ Made me much more skeptical.
- ○ Made me somewhat more skeptical.
- ○ Made me slightly more skeptical.
- ○ Has not moved me in either direction.
- ○ Made me slightly more confident.
- ○ Made me somewhat more confident.
- ○ Made me much more confident.

Next >> Exit and clear survey

Questionnaire, page 23: Transition text to socio-demographics

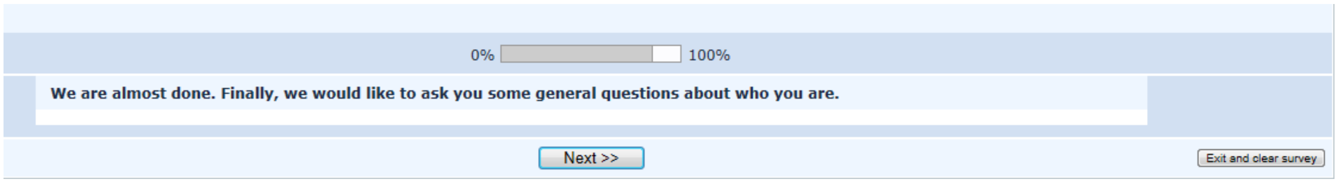

Questionnaire, page 24: Socio-demographics I

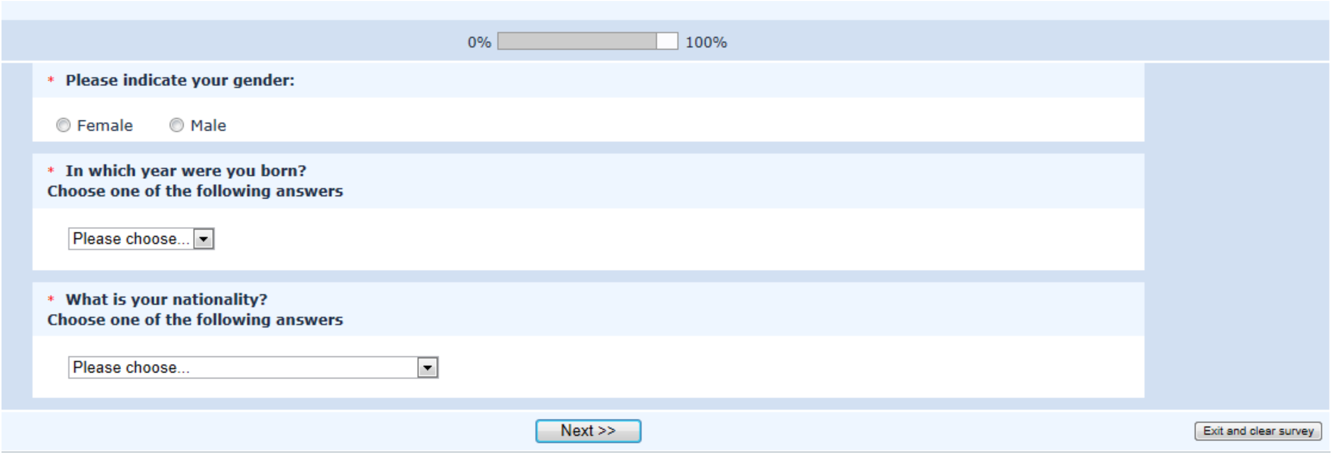

Questionnaire, page 25: Socio-demographics II

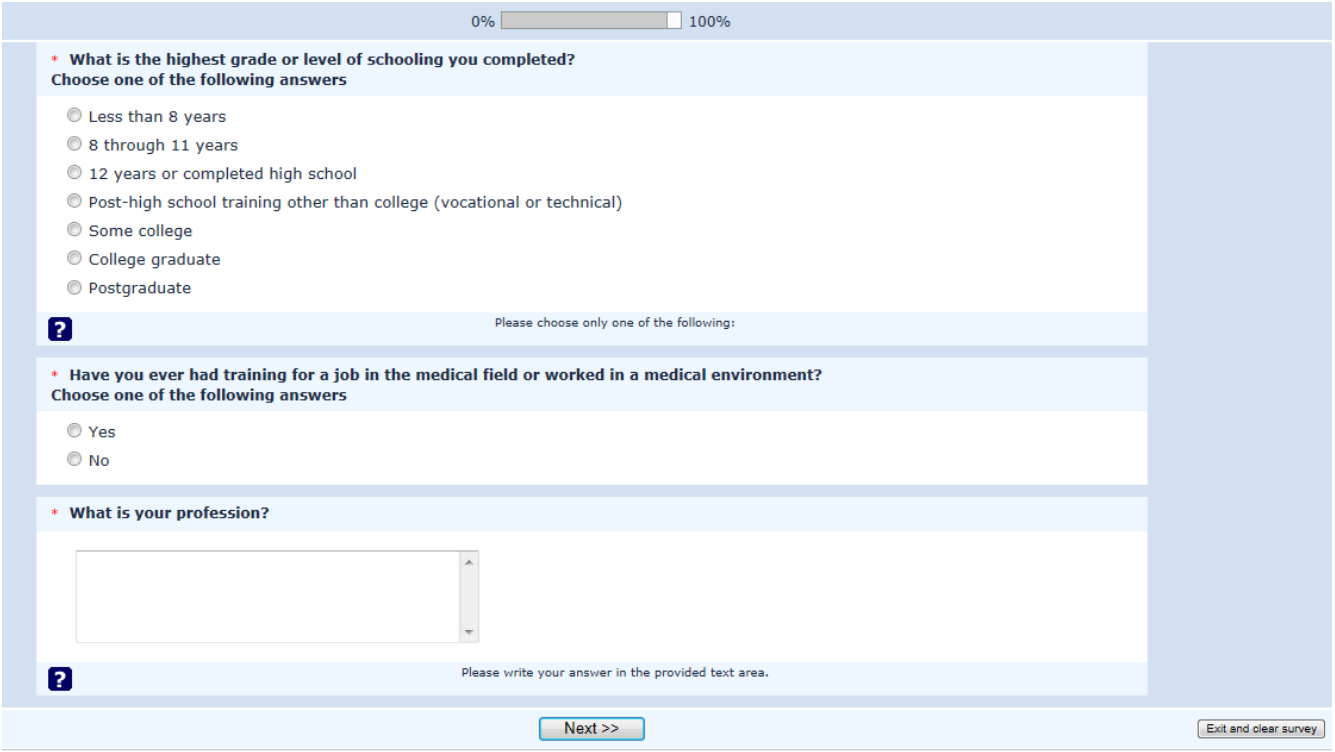

0% 100%

* **What is the highest grade or level of schooling you completed?**
Choose one of the following answers

- Less than 8 years
- 8 through 11 years
- 12 years or completed high school
- Post-high school training other than college (vocational or technical)
- Some college
- College graduate
- Postgraduate

? Please choose only one of the following:

* **Have you ever had training for a job in the medical field or worked in a medical environment?**
Choose one of the following answers

- Yes
- No

* **What is your profession?**

? Please write your answer in the provided text area.

Next >> Exit and clear survey

Questionnaire, page 26: Socio-demographics III

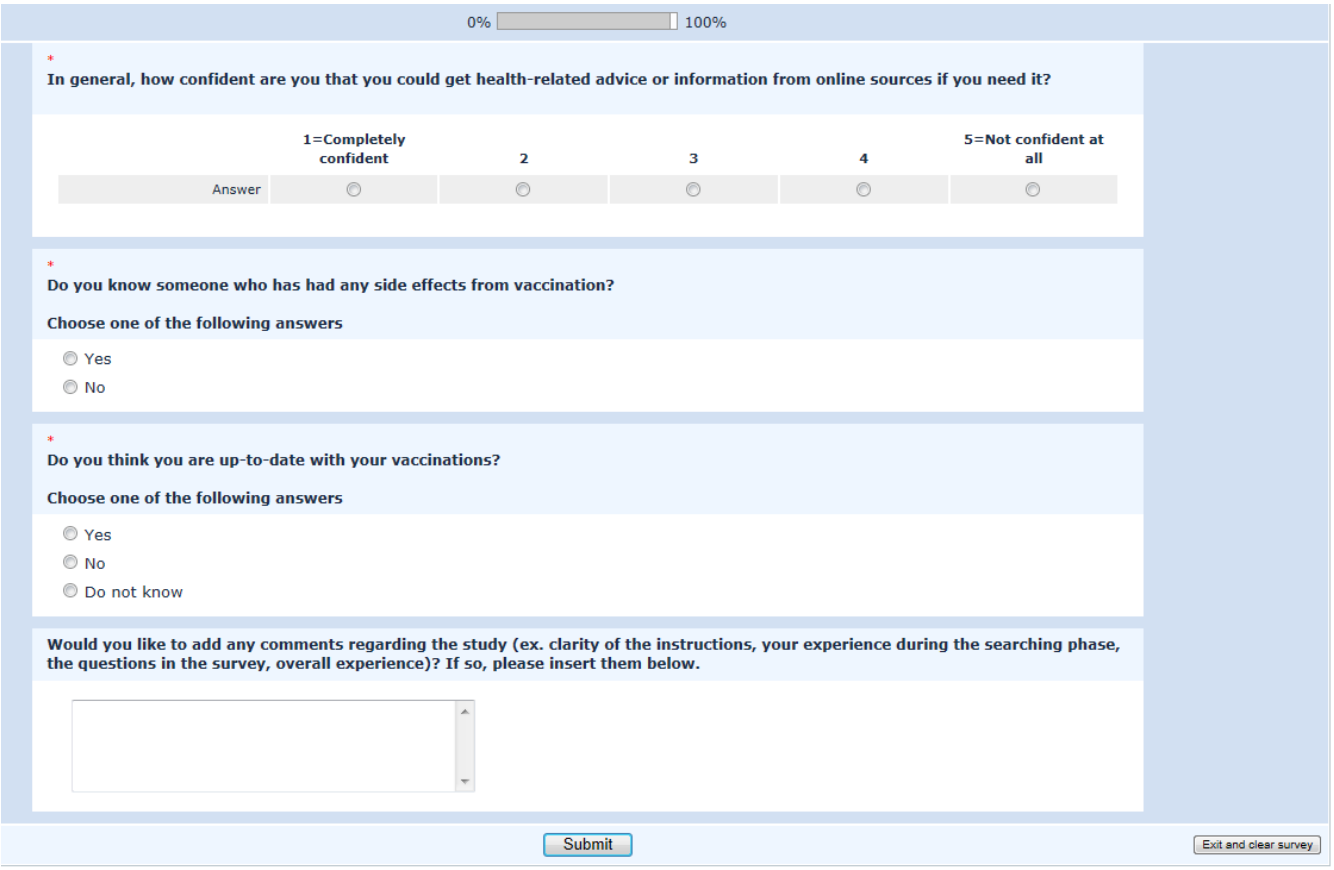
0% 100%

*
In general, how confident are you that you could get health-related advice or information from online sources if you need it?

	1=Completely confident	2	3	4	5=Not confident at all
Answer	○	○	○	○	○

*
Do you know someone who has had any side effects from vaccination?

Choose one of the following answers

- ○ Yes
- ○ No

*
Do you think you are up-to-date with your vaccinations?

Choose one of the following answers

- ○ Yes
- ○ No
- ○ Do not know

Would you like to add any comments regarding the study (ex. clarity of the instructions, your experience during the searching phase, the questions in the survey, overall experience)? If so, please insert them below.

Submit

Exit and clear survey
