# Supplementary material for: Manipulating Google’s Knowledge Graph Box to Counter Biased Information Processing During an Online Search on Vaccination: Application of a Technological Debiasing Strategy
Source: J Med Internet Res. 2016 Jun 2;18(6):e137. doi: 10.2196/jmir.5430 (PMC4911515; doi:10.2196/jmir.5430)
Supplement: Multimedia Appendix 5 [file jmir_v18i6e137_app5.pdf]

Age

Experimental group	N	Mean	Std. Deviation	Std. Error	95% Confidence Interval for Mean		Minimum	Maximum
					Lower Bound	Upper Bound		
Group 1	45	38.31	11.481	1.711	34.86	41.76	19	64
Group 2	45	39.02	10.382	1.548	35.90	42.14	22	69
Group 3	46	36.43	9.919	1.462	33.49	39.38	20	57
Group 4	50	37.30	10.403	1.471	34.34	40.26	19	58
Group 5	47	35.15	9.580	1.397	32.34	37.96	22	61
Group 6	46	37.91	12.229	1.803	34.28	41.54	19	67
Total	279	37.34	10.674	.639	36.08	38.59	19	69

ANOVA

Age

	Sum of Squares	df	Mean Square	F	Sig.
Between Groups	448.294	5	89.659	.784	.562
Within Groups	31224.036	273	114.374		
Total	31672.330	278			

			Experimental group						Total
			Group 1	Group 2	Group 3	Group 4	Group 5	Group 6	
Nationality	United States	Count	20	17	26	21	16	21	121
		Expected Count	19.5	19.5	19.9	21.7	20.4	19.9	121.0
	United Kingdom	Count	14	16	10	16	14	12	82
		Expected Count	13.2	13.2	13.5	14.7	13.8	13.5	82.0
	Canada	Count	9	9	5	7	14	8	52
		Expected Count	8.4	8.4	8.6	9.3	8.8	8.6	52.0
	Australia	Count	0	1	0	2	1	1	5
		Expected Count	.8	.8	.8	.9	.8	.8	5.0
	New Zealand	Count	0	1	2	0	0	0	3
		Expected Count	.5	.5	.5	.5	.5	.5	3.0
	Other	Count	2	1	3	4	2	4	16
		Expected Count	2.6	2.6	2.6	2.9	2.7	2.6	16.0
Total		Count	45	45	46	50	47	46	279
		Expected Count	45.0	45.0	46.0	50.0	47.0	46.0	279.0

Chi-Square Tests

	Value	df	Asymp. Sig. (2-sided)	Monte Carlo Sig. (2-sided)		
				Sig.	99% Confidence Interval	
					Lower Bound	Upper Bound
Pearson Chi-Square	23.089[a]	25	.572	.590[b]	.578	.603
Likelihood Ratio	24.238	25	.506	.664[b]	.652	.676
Fisher's Exact Test	20.209			.657[b]	.644	.669
Linear-by-Linear Association	.083[c]	1	.774	.770[b]	.760	.781
N of Valid Cases	279					

a. 18 cells (50.0%) have expected count less than 5. The minimum expected count is .48.
b. Based on 10000 sampled tables with starting seed 2000000.
c. The standardized statistic is .287.

			Experimental group						Total
			Group 1	Group 2	Group 3	Group 4	Group 5	Group 6	
Education level	College graduate or postgraduate studies	Count	27	21	17	29	23	28	145
		Expected Count	23.4	23.4	23.9	26.0	24.4	23.9	145.0
	Some college level	Count	11	9	11	10	12	9	62
		Expected Count	10.0	10.0	10.2	11.1	10.4	10.2	62.0
	Post high school vocational or technical training	Count	0	3	5	2	5	1	16
		Expected Count	2.6	2.6	2.6	2.9	2.7	2.6	16.0
	Between 8 to 12 years or completed high school degree	Count	7	12	13	9	7	8	56
		Expected Count	9.0	9.0	9.2	10.0	9.4	9.2	56.0
Total		Count	45	45	46	50	47	46	279
		Expected Count	45.0	45.0	46.0	50.0	47.0	46.0	279.0

Chi-Square Tests						
	Value	df	Asymp. Sig. (2-sided)	Monte Carlo Sig. (2-sided)		
				Sig.	99% Confidence Interval	
					Lower Bound	Upper Bound
Pearson Chi-Square	16.562[a]	15	.346	.348[b]	.336	.361
Likelihood Ratio	18.648	15	.230	.283[b]	.272	.295
Fisher's Exact Test	16.215			.345[b]	.332	.357
Linear-by-Linear Association	.401[c]	1	.527	.529[b]	.516	.542
N of Valid Cases	279					

a. 6 cells (25.0%) have expected count less than 5. The minimum expected count is 2.58.
b. Based on 10000 sampled tables with starting seed 92208573.
c. The standardized statistic is -.633.

			Experimental group						Total
			Group 1	Group 2	Group 3	Group 4	Group 5	Group 6	
Have you ever had training for a job in the medical field or worked in a medical environment?	Yes	Count	7	2	4	5	4	6	28
		Expected Count	4.5	4.5	4.6	5.0	4.7	4.6	28.0
	No	Count	38	43	42	45	43	40	251
		Expected Count	40.5	40.5	41.4	45.0	42.3	41.4	251.0
Total		Count	45	45	46	50	47	46	279
		Expected Count	45.0	45.0	46.0	50.0	47.0	46.0	279.0

Chi-Square Tests

	Value	df	Asymp. Sig. (2-sided)	Monte Carlo Sig. (2-sided)		
				Sig.	99% Confidence Interval	
					Lower Bound	Upper Bound
Pearson Chi-Square	3.750[a]	5	.586	.604[b]	.592	.617
Likelihood Ratio	3.899	5	.564	.601[b]	.588	.613
Fisher's Exact Test	3.715			.602[b]	.589	.614
Linear-by-Linear Association	.001[c]	1	.977	1.000[b]	1.000	1.000
N of Valid Cases	279					

a. 5 cells (41.7%) have expected count less than 5. The minimum expected count is 4.52.
b. Based on 10000 sampled tables with starting seed 1993510611.
c. The standardized statistic is -.029.

In general, how confident are you that you could get health-related advice or information from online sources if you need it? [Scale from 1=Not confident at all to 5=Completely confident]								
	N	Mean	Std. Deviation	Std. Error	95% Confidence Interval for Mean		Minimum	Maximum
					Lower Bound	Upper Bound		
Group 1	45	3.58	1.011	.151	3.27	3.88	1	5
Group 2	45	3.78	1.064	.159	3.46	4.10	2	5
Group 3	46	3.63	.974	.144	3.34	3.92	1	5
Group 4	50	3.78	1.075	.152	3.47	4.09	2	5
Group 5	47	3.57	.927	.135	3.30	3.85	2	5
Group 6	46	3.70	1.008	.149	3.40	4.00	1	5
Total	279	3.67	1.006	.060	3.56	3.79	1	5

Chi-Square Tests						
	Value	df	Asymp. Sig. (2-sided)	Monte Carlo Sig. (2-sided)		
				Sig.	99% Confidence Interval	
					Lower Bound	Upper Bound
Pearson Chi-Square	3.048[a]	5	.693	.698[b]	.686	.710
Likelihood Ratio	3.074	5	.689	.707[b]	.696	.719
Fisher's Exact Test	3.029			.700[b]	.688	.712
Linear-by-Linear Association	.602[c]	1	.438	.463[b]	.451	.476
N of Valid Cases	279					

a. 4 cells (33.3%) have expected count less than 5. The minimum expected count is 4.84.
b. Based on 10000 sampled tables with starting seed 79654295.
c. The standardized statistic is .776.

ANOVA

	Sum of Squares	df	Mean Square	F	Sig.
Between Groups	2.038	5	.408	.398	.850
Within Groups	279.281	273	1.023		
Total	281.319	278			
