# Supplementary material for: Manipulating Google’s Knowledge Graph Box to Counter Biased Information Processing During an Online Search on Vaccination: Application of a Technological Debiasing Strategy
Source: J Med Internet Res. 2016 Jun 2;18(6):e137. doi: 10.2196/jmir.5430 (PMC4911515; doi:10.2196/jmir.5430)
Supplement: Multimedia Appendix 6 [file jmir_v18i6e137_app6.pdf]

The following heat maps were generated using all registered cursor movements for each group during the whole experiment.

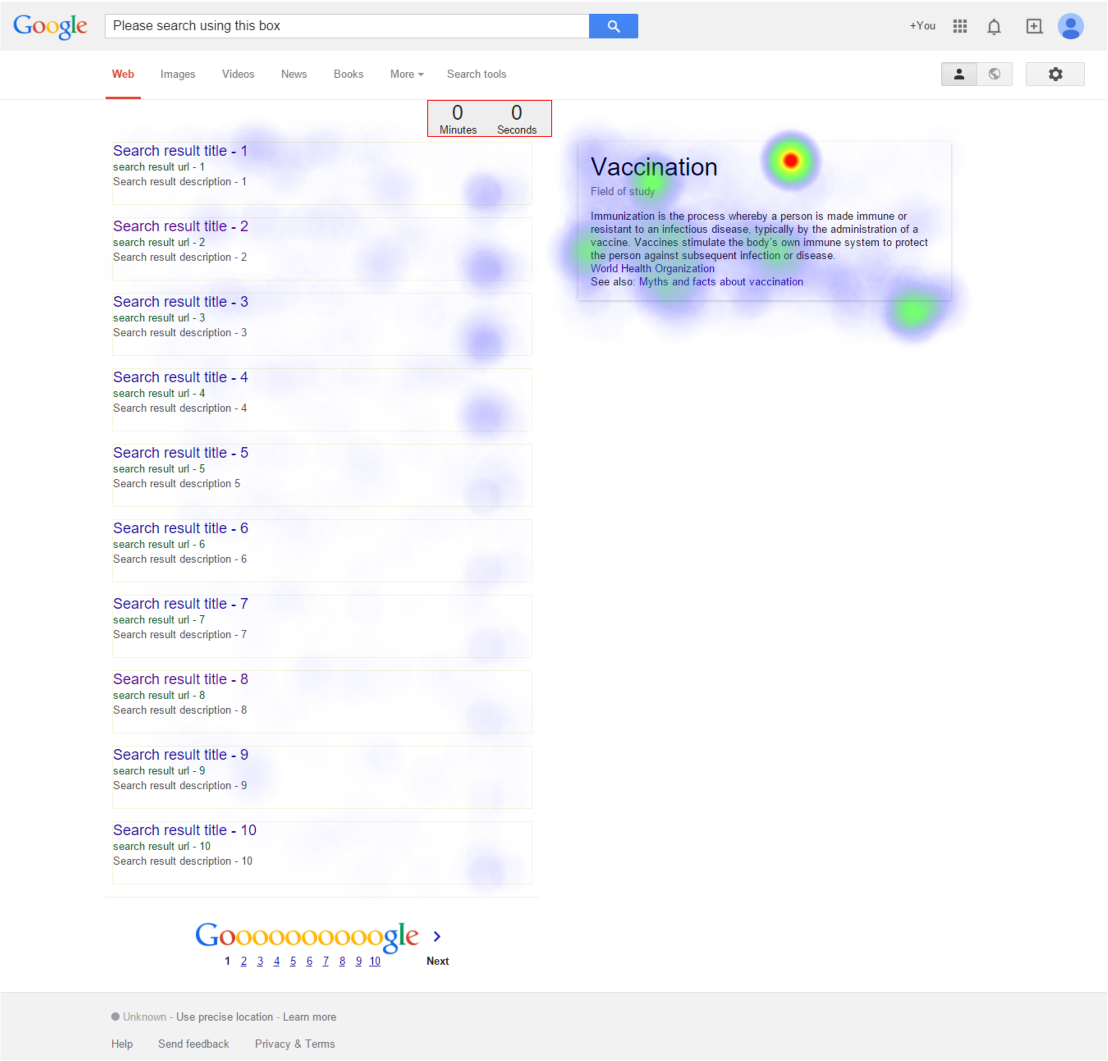

Comprehensible basic information [WHO], Group 1

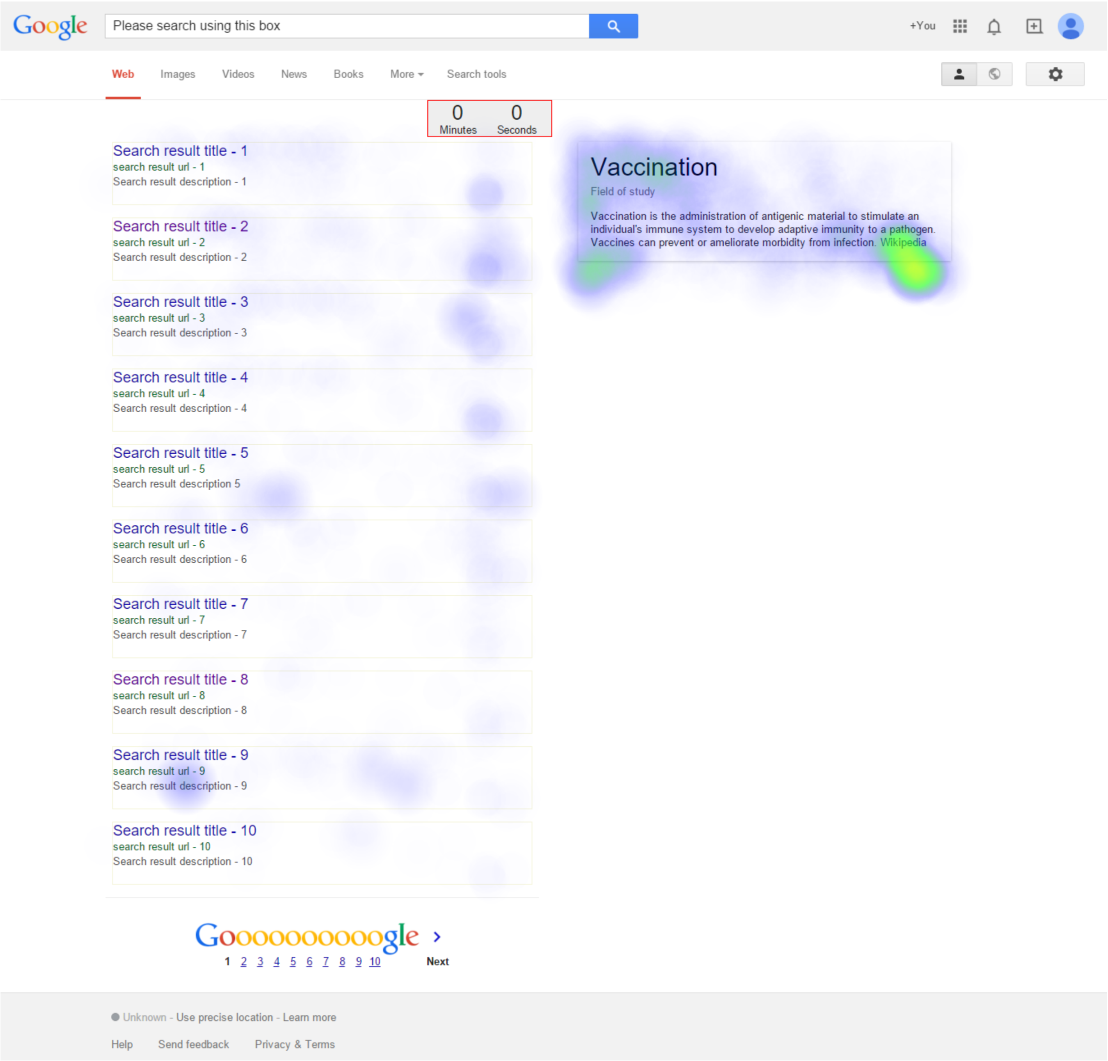

Hardly comprehensible basic information [Wikipedia], Group 2

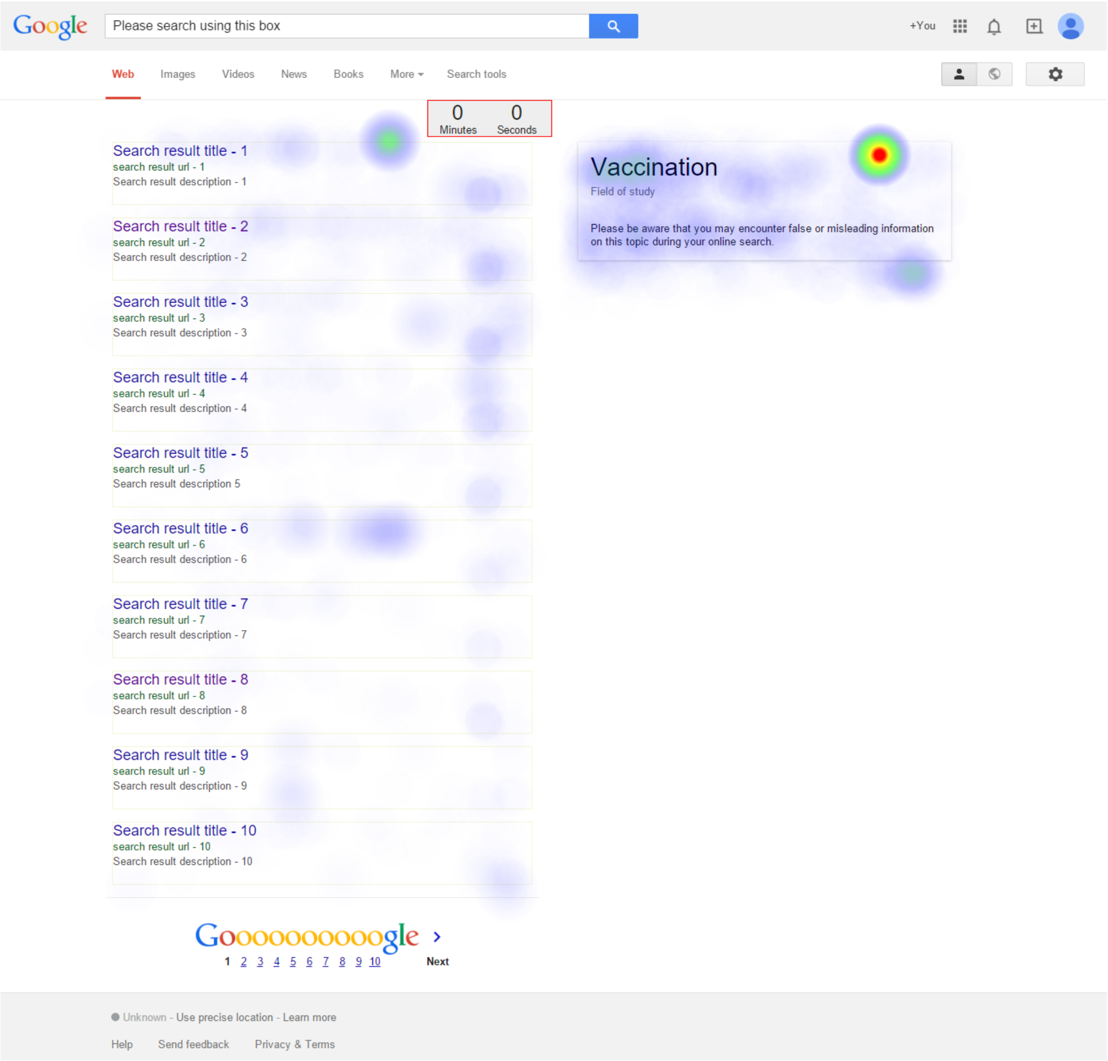

Warning present, Group 3

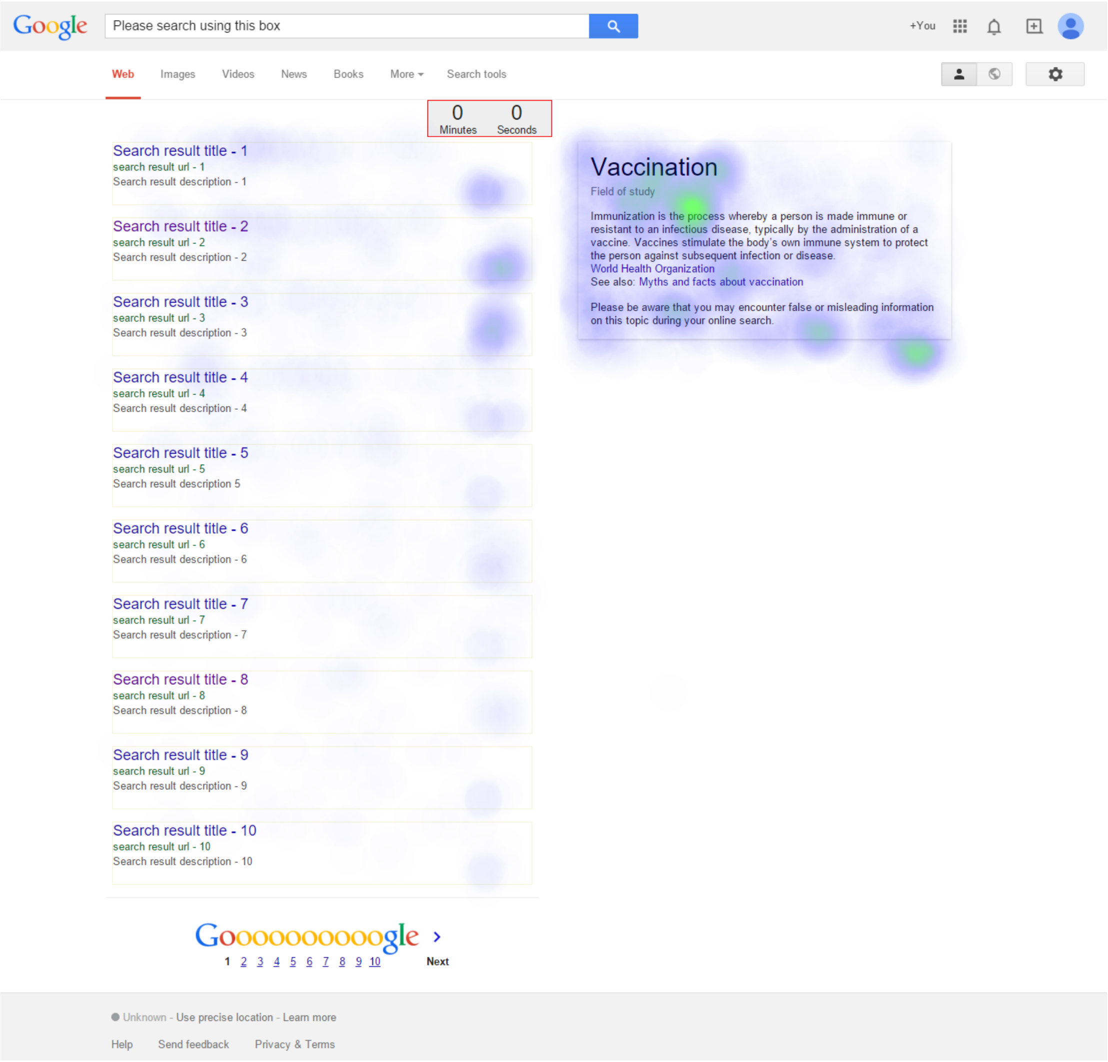

Comprehensible basic information [WHO] plus warning present, Group 4

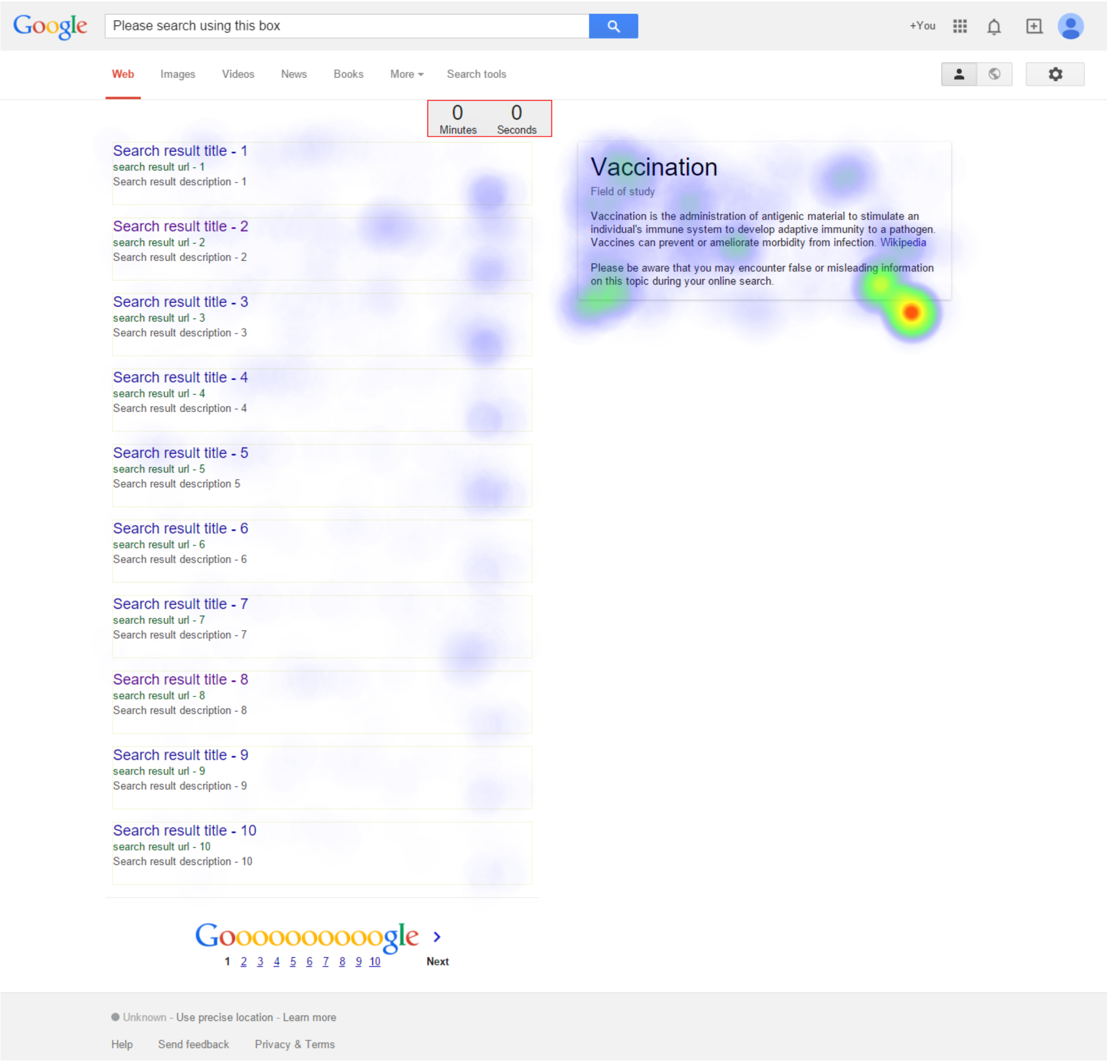

Hardly comprehensible basic information [Wikipedia] plus warning present, Group 5

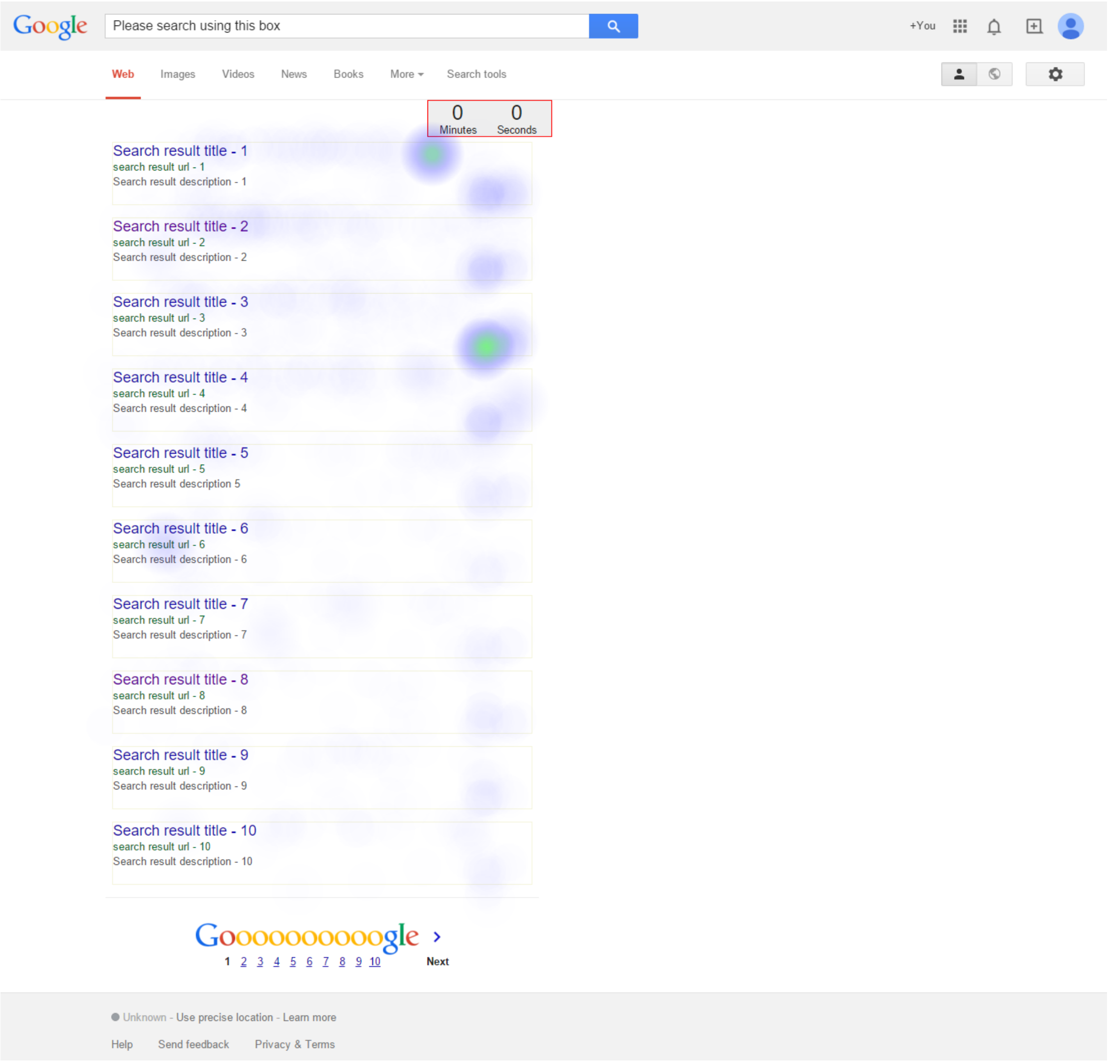

Control group, Group 6
